# Regulation of branched versus linear Arp2/3‐generated actin filaments

**DOI:** 10.15252/embj.2022113008

**Published:** 2023-03-20

**Authors:** Luyan Cao, Foad Ghasemi, Michael Way, Antoine Jégou, Guillaume Romet‐Lemonne

**Affiliations:** ^1^ Université Paris Cité, CNRS, Institut Jacques Monod Paris France; ^2^ The Francis Crick Institute London UK; ^3^ Department of Infectious Disease Imperial College London UK

**Keywords:** actin filament turnover, Arp2/3 complex, cytoskeleton, microfluidics, WISH/DIP/SPIN90, Cell Adhesion, Polarity & Cytoskeleton

## Abstract

Activation of the Arp2/3 complex by VCA‐motif‐bearing actin nucleation‐promoting factors results in the formation of “daughter” actin filaments branching off the sides of pre‐existing “mother” filaments. Alternatively, when stimulated by SPIN90, Arp2/3 directly nucleates “linear” actin filaments. Uncovering the similarities and differences between these two mechanisms is fundamental to understanding how actin cytoskeleton dynamics are regulated. Here, analysis of individual filaments reveals that, unexpectedly, the VCA motifs of WASP, N‐WASP, and WASH destabilize existing branches, as well as SPIN90‐Arp2/3 at linear filament ends. Furthermore, branch stabilizer cortactin and destabilizer GMF each have a similar impact on SPIN90‐activated Arp2/3. However, unlike branch junctions, SPIN90‐Arp2/3 at the ends of linear filaments is not destabilized by piconewton forces and does not become less stable with time. It thus appears that linear and branched Arp2/3‐generated filaments respond similarly to the regulatory proteins we have tested, albeit with some differences, but significantly differ in their responses to aging and mechanical stress. These kinetic differences likely reflect the small conformational differences recently reported between Arp2/3 in branch junctions and linear filaments and suggest that their turnover in cells may be differently regulated.

## Graphical abstract

Linear and branched Arp2/3‐generated actin filaments respond differently to aging and mechanical stress, but are regulated in a similar manner by actin regulatory proteins *in vitro*.

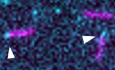

## Introduction

Branched actin filament networks, such as the ones found in the lamellipodia of migrating cells, or endocytic patches, are a hallmark example of dynamic actin networks in cells (Lappalainen *et al*, 2022). Branches result from the activation of the Arp2/3 complex by the VCA motifs of nucleation‐promoting factors (NPFs), such as WASP or WAVE (See Gautreau *et al* (2022) for a review). The VCA motif binds to the Arp2/3 complex via its C and A domains, and recruits an actin monomer (G‐actin) via its V‐domain (also known as WH2 domain). Two VCA motifs can bind to the same Arp2/3 complex, and activate it more efficiently (Padrick *et al*, 2011; Zimmet *et al*, 2020). Following the binding of this large complex to the side of a pre‐existing filament, commonly called the “mother” filament, the VCA motifs detach, thereby allowing the elongation of the branch (Smith *et al*, 2013). Actin subunits are added at the dynamic “barbed” end of the “branches,” while their “pointed” end is stabilized and connected to the mother filament by the Arp2/3 complex.

In addition to forming branches, the Arp2/3 complex can also be activated to nucleate linear filaments by binding the protein WISH/DIP/SPIN90, hereafter referred to as SPIN90 (Wagner *et al*, 2013). It results in the nucleation of an actin filament that elongates freely at the barbed end, while SPIN90‐Arp2/3 remains attached to the pointed end. This activation mechanism generates *de novo* linear filaments in the absence of a pre‐existing mother filament.

These two nucleation mechanisms raise an immediate question: are the two activated states of Arp2/3 identical? Recent high‐resolution cryo‐EM structures of Arp2/3 bound to SPIN90 (dip1 from yeast), and of Arp2/3 at branch junctions in cells and *in vitro* already provide some answers (Fäßler *et al*, 2020; Shaaban *et al*, 2020; Ding *et al*, 2022). They indicate that Arp2/3 activation follows the same general mechanism in both cases, with some small differences in inter‐subunit contacts and in the extent to which Arp2 and Arp3 are displaced and adopt “flatter” conformations. Whether these small conformational differences alter interactions with regulatory proteins is an important fundamental question. Recent biochemical data suggest that this may indeed be the case, as formin mDia1 can bind to the SPIN90‐activated Arp2/3 and does not appear to bind to the Arp2/3 complex in nascent branches (Cao *et al*, 2020). Yet, it remains largely unknown how the two forms of activated Arp2/3 complex may differ in their response to regulatory factors.

A better understanding of this question would shed light on how branched and linear Arp2/3‐nucleated filaments can be mutually regulated in cells. Intriguingly, it appears that the two activation machineries of the Arp2/3 complex can synergize or compete, depending on the context. During endocytosis in yeast, linear SPIN90‐nucleated filaments provide the initial mother filaments that will “prime” the formation of branched networks (Basu & Chang, 2011; Balzer *et al*, 2019, 2020). This appears to be favored by the fact that dip1 and wsp1, the fission yeast homologs of SPIN90 and WASP, respectively, can simultaneously bind and co‐activate the Arp2/3 complex, to generate linear filaments (Balzer *et al*, 2020). In contrast, in the cortex of animal cells, SPIN90‐induced nucleation decreases the branching density and favors the rapid elongation of filaments by formin mDia1 (Cao *et al*, 2020).

To understand the reorganization and disassembly of actin filament networks in cells, an important aspect is the control of the stability of Arp2/3‐generated filaments. It has been studied in the case of branches but remains mostly unknown for linear filaments. With time, branches detach from mother filaments, as debranching is favored by the hydrolysis of ATP within the Arp2/3 complex (Le Clainche *et al*, 2003; Dayel & Mullins, 2004). Debranching can be modulated by proteins interacting with Arp2/3: branch junctions are stabilized by cortactin and destabilized by GMF (Weaver *et al*, 2001; Gandhi *et al*, 2010). In addition, debranching is accelerated by mechanical tension applied to the branch (Pandit *et al*, 2020). In contrast, while yeast SPIN90‐Arp2/3 has been observed to remain at the pointed ends of actin filaments for minutes (Balzer *et al*, 2019), several questions remain unanswered. Is the pointed end eventually uncapped or does Arp2/3 remain bound to it when SPIN90 departs? How long does this take? How is it impacted by regulatory factors?

Here, we address these questions using purified mammalian proteins in single‐filament experiments, using microfluidics and TIRF microscopy. We show that Arp2/3 at the filament pointed end is similarly stabilized by cortactin and destabilized by GMF, when activated either by VCA or SPIN90 to generate branches or linear filaments, respectively. Yet key differences, in particular, regarding their sensitivity to mechanical stress and Arp2/3 aging, indicate that the two activated states of Arp2/3 could be regulated independently in cells.

## Results

Experiments were performed at 25°C, using actin from rabbit skeletal muscle, Arp2/3 from bovine brain, and recombinant mammalian isoforms of other proteins (see Materials and Methods).

### VCA enhances the nucleation of linear filaments by SPIN90‐Arp2/3

**Figure 1:**
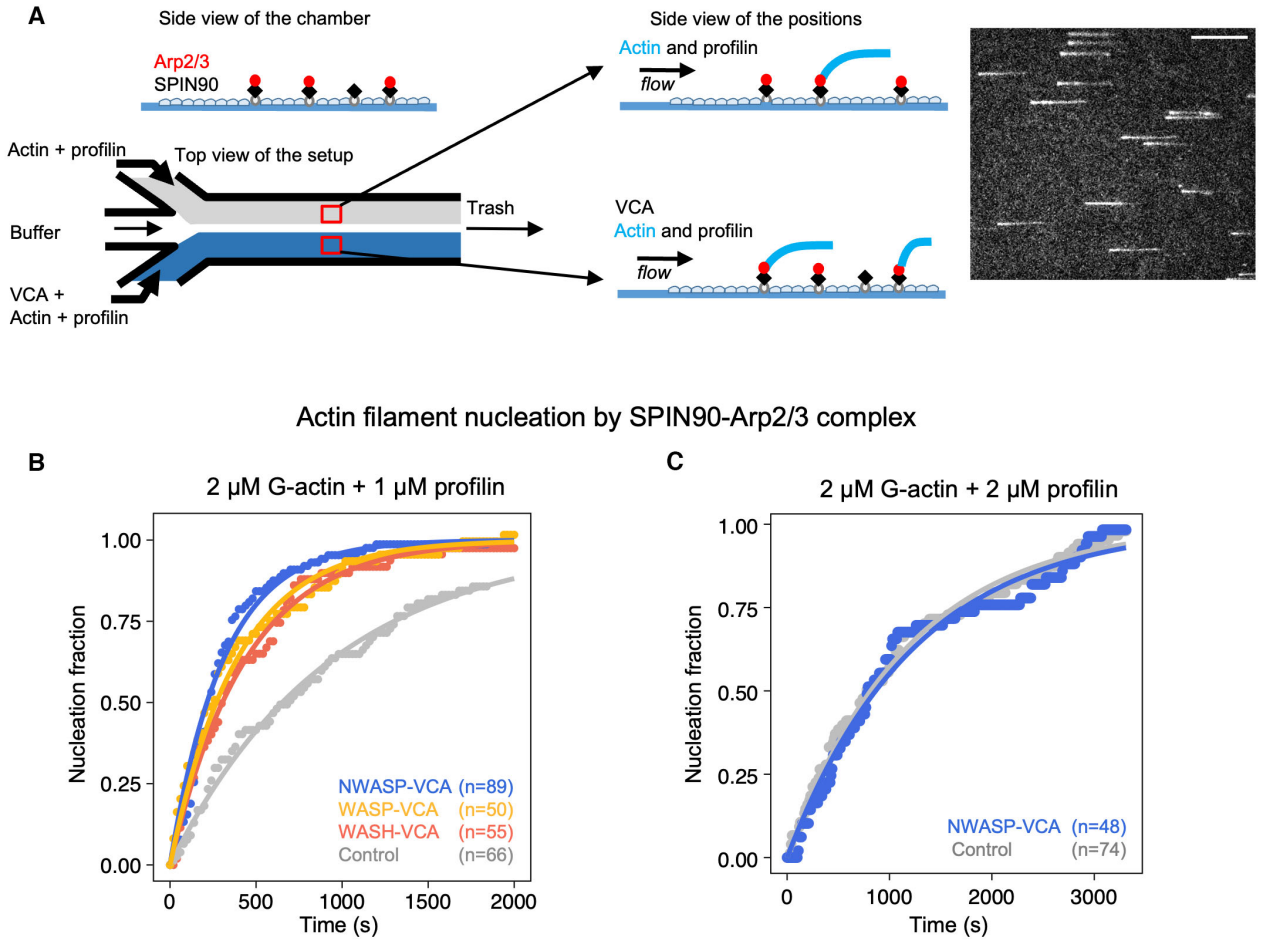
Mammalian VCA motifs accelerate the nucleation of filaments by SPIN90‐Arp2/3 Schematic of the nucleation experiment. In a microfluidics chamber, SPIN90 is attached to the coverslip surface via its His‐tag, and exposed to Arp2/3. Half of the chamber is then exposed to actin and profilin, while the other half is exposed to actin, profilin, and VCA. Actin is labeled with Alexa488 (15%), and one can monitor filaments as they nucleate and grow from the surface. Right: TIRF microscopy image of actin filaments. Scale bar: 20 μm.Normalized number of filaments nucleated over time, from SPIN90‐Arp2/3 exposed to 2 μΜ G‐actin (15% labeled with Alexa488) and 1 μΜ profilin, with 0 or 0.5 μM of GST‐VCA from different NPFs. Solid lines are exponential fits, yielding nucleation rates k_nuc_ = (1.06 ± 0.03) × 10^−3^ s^−1^, without VCA, and k_nuc_ = (3.23 ± 0.08) × 10^−3^, (2.58 ± 0.05) × 10^−3^, and (2.28 ± 0.03) × 10^−3^ s^−1^ with VCA from N‐WASP, WASP, and WASH, respectively.Same as in (B), with 2 μM G‐actin and 2 μM profilin. The exponential fits yield k_nuc_ = (8.5 ± 0.1) × 10^−4^ s^−1^ without VCA, and k_nuc_ = (8.0 ± 0.2) × 10^−4^ s^−1^ with 0.5 μM GST‐VCA from N‐WASP. Data information: In (B, C), indicated values of n are the number of filaments observed in each individual experiment. These experiments were repeated three times, with similar numbers of filaments in each experiment, yielding similar results. Source data are available online for this figure.

We first sought to verify that, as with their yeast homologs (Balzer *et al*, 2020), mammalian VCA and SPIN90 can co‐activate the Arp2/3 complex to generate linear filaments. To do so, we exposed surface‐anchored SPIN90 to the Arp2/3 complex, and subsequently flowed in solutions of profilin‐actin complex and different concentrations of VCA into the microfluidics chamber (Fig 1A). Since the outcome of these experiments is very sensitive to small variations in protein concentration, we have taken advantage of a microfluidics assay to simultaneously perform experiments and controls in two different regions of the same microchamber to minimize variations between experiments (see Materials and Methods, Fig 1A; Jégou *et al*, 2011; Wioland *et al*, 2020, 2022).

We found that the presence of VCA motifs from WASP, N‐WASP, or WASH, all induce faster nucleation of filaments from SPIN90‐Arp2/3 (Fig 1B). VCA from N‐WASP appeared to be slightly more potent than VCA from WASP and WASH in enhancing nucleation from SPIN90‐Arp2/3 (with 2 μM G‐actin and 1 μM profilin, the estimated nucleation rate is 25–40% higher). We wondered if the VCA motifs from these three NPFs ranked similarly for the activation of Arp2/3‐induced branching. We thus compared the branch densities on pre‐formed filaments, 90 s after exposing them for 90 s to Arp2/3, G‐actin, and VCA from the different NPFs (see Materials and Methods). We observed the same ranking, with a more pronounced difference between NPFs: the branch density was 0.95, 0.43, and 0.27 branches/μm with VCA motifs from N‐WASP, WASP, and WASH, respectively.

VCA recruits G‐actin as part of the branched nucleation process (Chereau *et al*, 2005). With yeast homologs, it was found that the co‐activation of Arp2/3 by VCA to form linear filaments required the recruitment of G‐actin by VCA (Balzer *et al*, 2020). We thus hypothesized that the availability of G‐actin would impact the ability of mammalian VCA to enhance the nucleation of filaments from SPIN90‐Arp2/3. To test this hypothesis, we took advantage of profilin, which competes with VCA for G‐actin. We found that, in the presence of equimolar amounts of profilin and actin, VCA from N‐WASP no longer enhanced the nucleation of filaments from SPIN90‐Arp2/3 (Fig 1C). This observation confirms that VCA enhances SPIN90‐induced nucleation by recruiting actin monomers.

### VCA motifs destabilize Arp2/3 at the pointed end of branches and linear filaments

During the course of our experiments, we also noticed that the SPIN90‐Arp2/3‐nucleated filaments detached faster from the surface in the presence of VCA (Appendix Fig S1). This indicates that VCA destabilizes SPIN90‐Arp2/3 at pointed ends. To obtain further insights into this destabilization mechanism, we decided to quantify VCA‐mediated detachment of SPIN90‐Arp2/3‐nucleated filaments in the presence of 0.15 μM G‐actin (to maintain filaments at a constant length).

**Figure EV1:**
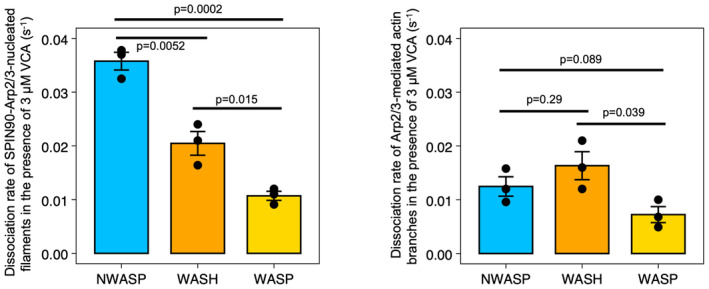
Comparing the VCA motifs from different NPFs in their ability to destabilize linear filaments nucleated by SPIN90‐Arp2/3 (left) or Arp2/3‐mediated branches (right) The dissociation rates of Arp2/3‐nucleated filaments exposed to nearly saturating amounts (3 μM) of VCA from different NPFs were measured, as in Fig 2. The histogram represents the average koff of three independent measurements (black dots) and the error bars represent the standard deviation. The dissociation rate of SPIN90‐Arp2/3‐nucleated filaments is 0.037 ± 0.003, 0.022 ± 0.002, and 0.011 ± 0.001 s^−1^, for N‐WASP, WASH, and WASP, respectively. The dissociation rate of Arp2/3‐mediated actin branches is 0.012 ± 0.003, 0.016 ± 0.005, and 0.007 ± 0.003 s^−1^, for N‐WASP, WASH, and WASP, respectively. The *P*‐values were determined with an unpaired *t*‐test.

**Figure 2:**
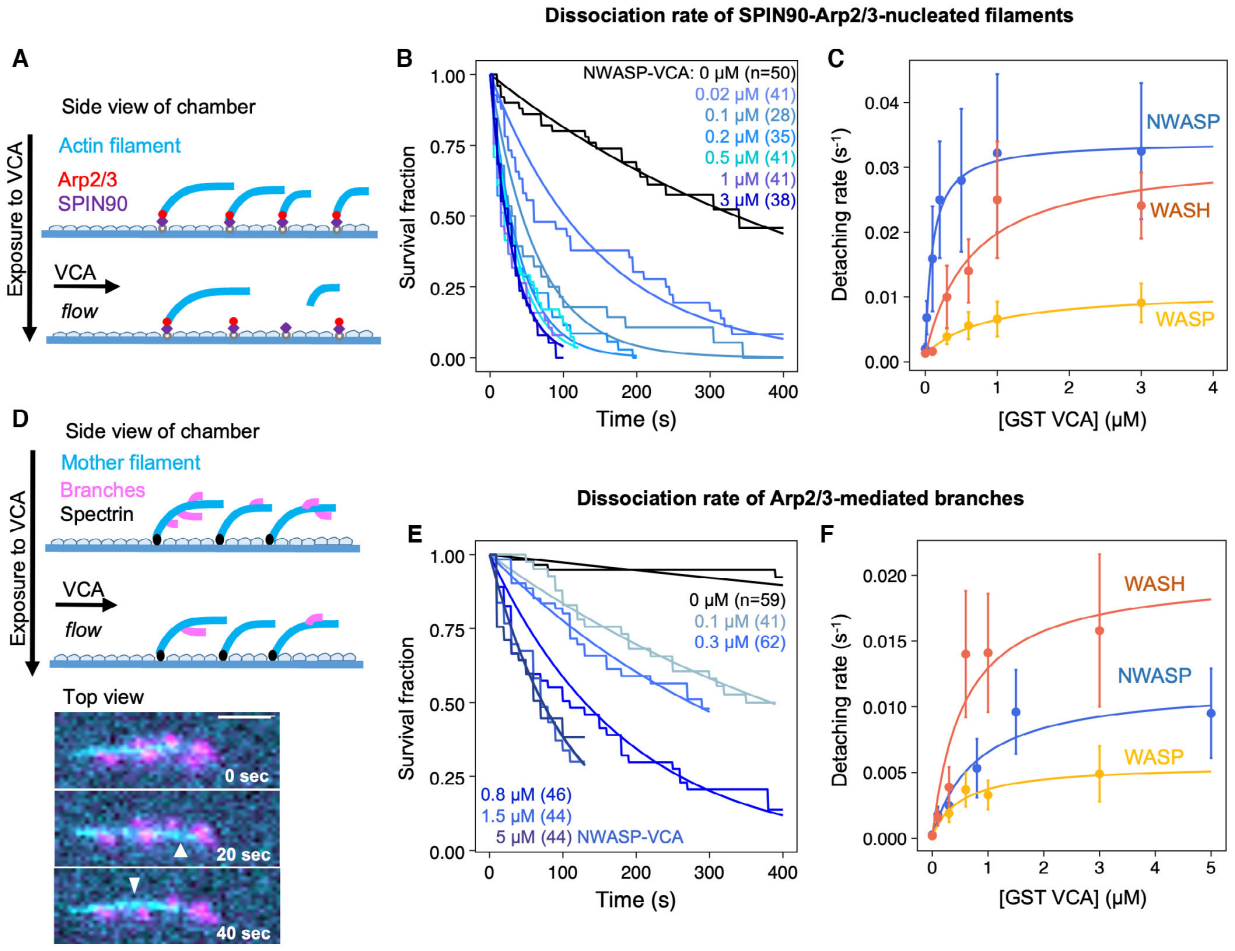
VCA accelerates the dissociation of both SPIN90‐nucleated actin filaments and branches A–CDissociation of SPIN90‐Arp2/3‐nucleated filaments. (A) Schematic of the detachment experiment. Actin filaments were first nucleated from a SPIN90‐Arp2/3‐decorated surface and elongated. Buffer with different concentrations of VCA was flowed in, and filament detachment events were monitored over time. (B) The fraction of filaments still attached to the surface, versus time, for different concentrations of GST‐NWASP‐VCA. Black lines are exponential fits. (C) Detachment rates determined by exponential fits of survival curves (as in B) as a function of the concentration of VCA motifs from different NPFs. The data are fitted by a Michaelis–Menten equation (resulting in an apparent K_D_ of 0.10 ± 0.01, 0.65 ± 0.36, and 0.82 ± 0.14 μM, for N‐WASP, WASH, and WASP, respectively; and a maximum detachment rate of 0.033 ± 0.001, 0.031 ± 0.006, and 0.010 ± 0.001 s^−1^ for WASH, N‐WASP, and WASP, respectively).D–FDissociation of Arp2/3‐mediated branches. (D) Schematic of the debranching experiment. Alexa488(15%)‐actin filaments (cyan) were elongated from surface‐anchored spectrin‐actin seeds, then exposed to VCA, Arp2/3, and Alexa568(15%)‐actin to form branches (magenta). After 5 min, buffer with different concentrations of VCA was flowed in, and debranching events were monitored over time. Bottom: fluorescence microscopy images, showing debranching event (white arrowheads). Scale bar: 3 μm. (E) The fraction of remaining branches, versus time, for different concentrations of GST‐NWASP‐VCA. Black lines are exponential fits. (F) Debranching rates, determined by exponential fits of survival curves (as in E) as a function of the concentration of VCA motifs from different NPFs. The data are fitted by a Michaelis–Menten equation (resulting in an apparent K_D_ of 0.57 ± 0.35, 0.81 ± 0.36, and 0.53 ± 0.23 μM, for WASH, N‐WASP, and WASP, respectively; and a maximum detachment rate of 0.020 ± 0.004, 0.012 ± 0.002, and 0.005 ± 0.001 s^−1^ for WASH, N‐WASP, and WASP, respectively). Data information: Error bars in (C, F) result from fits of the extremes of the 95% confidence intervals of survival functions (not shown in (B, E) for readability). The curves shown in (B, E) are each the result of one individual experiment carried out with NWASP‐VCA. They were repeated twice (technical replicates), for all three VCA motifs, yielding similar results. The results of three repeats at 3 μM of different VCA motifs are compared in Fig EV1. Source data are available online for this figure.

Taking advantage of the sequential exposure provided by microfluidics, we elongated filaments from surface‐anchored SPIN90‐Arp2/3, and subsequently monitored their detachment, over time, as we exposed them to different concentrations of VCA motifs (Fig 2A–C). We found that all VCA motifs accelerated the detachment of filaments from the surface, in a dose‐dependent manner, albeit to different extents. Interestingly, the NPFs did not rank the same as for the enhancement of nucleation: VCA from N‐WASP was still the most effective (increasing the detachment rate up to 16‐fold in the 0–1 μM range) but WASP was much less effective than WASH (Fig EV1).

**Figure EV2:**
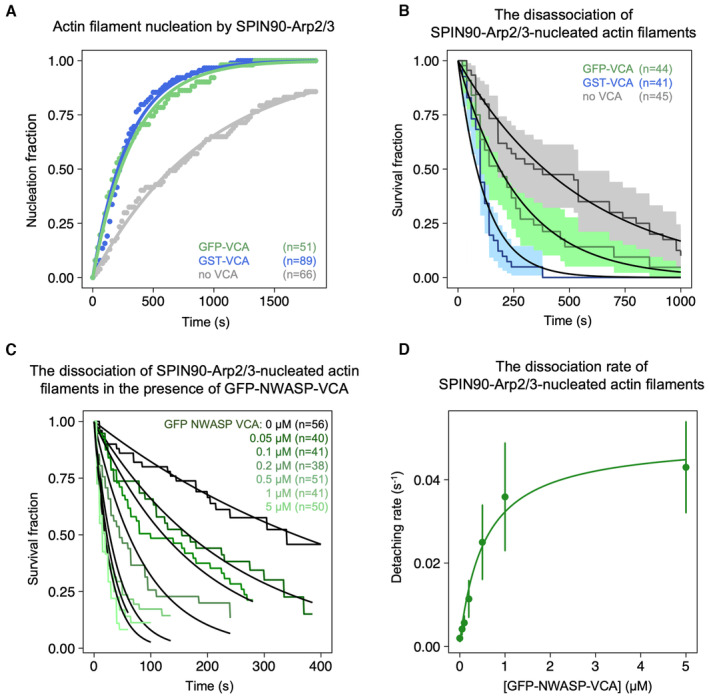
Impact of VCA dimerization Normalized number of filaments nucleated over time, from SPIN90‐Arp2/3 exposed to 2 μΜ G‐actin (15% labeled with Alexa488) and 1 μΜ profilin, with 0 or 0.5 μM of GST‐N‐WASP‐VCA or GFP‐N‐WASP‐VCA. Solid lines are exponential fits, yielding nucleation rates knuc = (1.06 ± 0.03) × 10^−3^ s^−1^ without VCA, and knuc = (3.23 ± 0.08) × 10^−3^ s^−1^ and (3.02 ± 0.04) × 10^−3^ s^−1^ with GST‐N‐WASP‐VCA and GFP‐N‐WASP‐VCA, respectively. Indicated values of *n* are the number of filaments observed in each experiment. These experiments were repeated three times, with similar results.Detachment of SPIN90‐Arp2/3‐nucleated filaments during the nucleation experiment shown in panel A. Solid lines are exponential fits, yielding dissociation rates koff = (1.8 ± 0.6) × 10^−3^ s^−1^ without VCA, and koff = (7.9 ± 1.9) × 10^−3^ s^−1^ and (3.7 ± 1.4) × 10^−3^ s^−1^ with GST‐N‐WASP‐VCA and with GFP‐N‐WASP‐VCA, respectively. Indicated values of N are the number of filaments observed in each experiment.The fraction of filaments still attached to the surface, versus time, for different concentrations of GFP‐NWASP‐VCA. Black lines are exponential fits. Indicated values of n are the number of filaments observed in each experiment. Each experiment was repeated twice (technical replicates), yielding similar results.Detachment rates determined by exponential fits of survival curves (in C) as a function of the concentration of VCA motifs from different NPFs. The error bars result from fits of the 95% confidence interval in the survival curves. The data are fitted by a Michaelis–Menten equation, resulting in KD = 0.56 ± 0.11 μM and Vmax = 0.049 ± 0.0034 s^−1^.

As dimerization enhances the branching activity of VCA motifs (Padrick *et al*, 2008), we decided to compare the effectiveness of dimerized GST‐ and monomeric GFP‐tagged N‐WASP VCA. We found that dimerization had no impact on the ability of VCA to accelerate the nucleation of filaments by SPIN90‐Arp2/3 (Fig EV2A). It did, however, enhance its ability to destabilize SPIN90‐Arp2/3 at the pointed end of the filaments: the apparent K_D_ of GFP‐VCA was 5.6‐fold higher than that of GST‐VCA (Fig EV2B–D).

To determine if VCA motifs could also destabilize Arp2/3‐mediated branches, we also performed similar experiments on branched filaments (Fig 2D–F). We found that all VCA motifs accelerated debranching, in a dose‐dependent manner. However, the NPFs did not rank the same as for the detachment of SPIN90‐Arp2/3‐nucleated filaments: VCA from WASP was still the least effective, but VCA from WASH was the most effective (accelerating debranching up to 83‐fold in the 0–3 μM range). Intriguingly, this ranking also differed from the branching situation, where we found VCA from WASH to be the least effective.

**Figure EV3:**
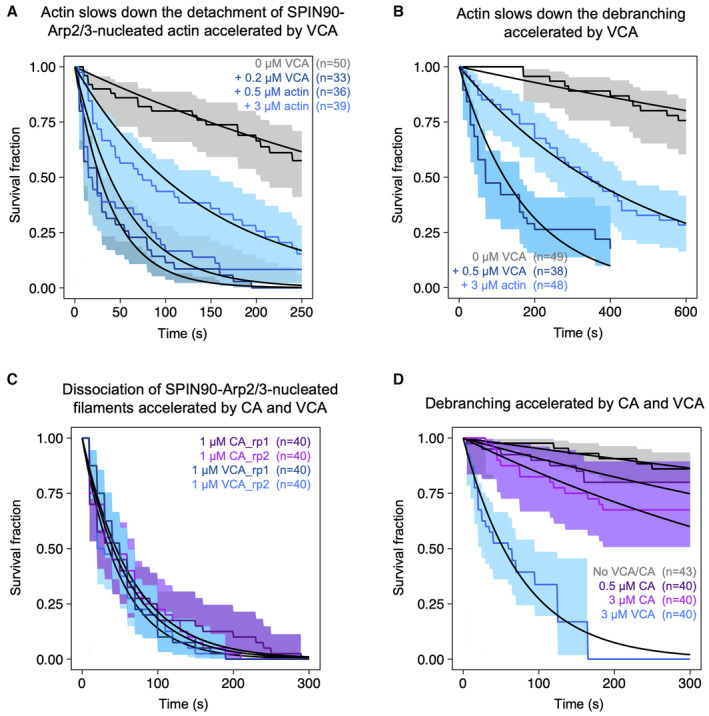
The presence of G‐actin inhibits the destabilizing action of VCA, but the V domain is not required to enhance the detachment of SPIN90‐Arp2/3‐nucleated filaments The fraction of filaments still attached to the surface versus time, exposed to buffer, or to 0.2 μΜ VCA with different concentrations of G‐actin. Black lines are exponential fits, yielding dissociation rates koff = (2.1 ± 0.8) × 10^−3^ S^−1^ without VCA, and koff = (2.6 ± 0.7) × 10^−2^ s^−1^ with 0.2 μΜ VCA, (1.8 ± 0.8) × 10^−2^ s^−1^ with 0.2 μΜ VCA plus 0.5 μΜ G‐actin, and (0.76 ± 0.28) × 10^−2^ s^−1^ with 0.2 μΜ VCA plus 3 μΜ G‐actin.The fraction of actin branches still attached to the mother filaments versus time, exposed to buffer, or to 0.5 μΜ VCA with 0.15 μM G‐actin, or to 0.5 μΜ VCA with 3 μΜ G‐actin. Black lines are exponential fits, yielding dissociation rates koff = (0.37 ± 0.7) × 10^−3^ s^−1^ without VCA, and koff = (5.8 ± 2.3) × 10^−3^ s^−1^ with 0.5 μΜ VCA, and (2.1 ± 0.7) × 10^−3^ s^−1^ with 0.5 μΜ VCA plus 3 μΜ G‐actin. The shaded areas represent 95% confidence intervals.The fraction of filaments still attached to the surface versus time, exposed to 0.2 μΜ G‐actin supplemented with 1 μΜ VCA or 1 μΜ CA. Black lines are exponential fits, yielding dissociation rates koff = (1.7 ± 0.02) × 10^−2^ s^−1^ and koff = (2.0 ± 0.02) × 10^−2^ s^−1^ for the two repeats with VCA; koff = (1.5 ± 0.02) × 10^−2^ s^−1^ and koff = (1.5 ± 0.02) × 10^−2^ s^−1^ for the two repeats with CA.The fraction of actin branches still attached to the mother filaments versus time, exposed to buffer, or to 0.15 μΜ actin supplemented with 0.5 μM CA, 3 μΜ CA, or 3 μΜ. Black lines are exponential fits, yielding dissociation rates koff = (5.0 ± 0.04) × 10^−4^ s^−1^ without buffer, and koff = (1.0 ± 0.01) × 10^−3^ s^−1^ with 0.5 μΜ CA, (1.8 ± 0.01) × 10^−3^ s^−1^ with 3 μΜ CA, and (1.3 ± 0.02) × 10^−2^ s^−1^ with 3 μΜ VCA. Data information: In the text above, uncertainty intervals correspond to the standard errors. In the figures, the shaded areas represent 95% confidence intervals. Indicated values of n are the number of filaments monitored in each experiment. The flowing solutions applied a weak force to the filaments (< 1 pN) and to the branches (0.2 pN).

Since the recruitment of G‐actin by VCA is necessary both for branching and for the enhancement of SPIN90‐induced nucleation (Chereau *et al*, 2005; Balzer *et al*, 2020; Fig 1C), we wondered how the availability of G‐actin would affect the ability of VCA to induce debranching and/or destabilize SPIN90‐Arp2/3 at filament pointed ends. We thus repeated the experiments in Fig 2 with different amounts of G‐actin. We found that the ability of VCA to destabilize Arp2/3, either at branch junctions or at the pointed end of linear filaments, was reduced by the presence of G‐actin (Fig EV3A and B). Since G‐actin can bind to the V‐domain of VCA, this suggests that the V‐domain may play a role in the destabilization of Arp2/3‐nucleated filaments.

To further investigate the role of the V‐domain, we repeated the experiments in Fig 2 with a construct comprising only the C and A domains from NWASP. We observed that this CA construct was as efficient as VCA at destabilizing SPIN90‐Arp2/3 at the pointed end of filaments, but that it was much less efficient than VCA at accelerating debranching (Fig EV3C and D). These results indicate that the V‐domain is entirely dispensable to destabilize SPIN90‐Arp2/3, while it seems to play an important role in the promotion of debranching. However, in the case of SPIN90‐Arp2/3‐nucleated filaments, it seems that having G‐actin bound to the V‐domain prevents the CA‐domain from interacting with the Arp2/3 complex.

### The debranching factor GMF destabilizes SPIN90‐Arp2/3‐nucleated filaments

**Figure 3:**
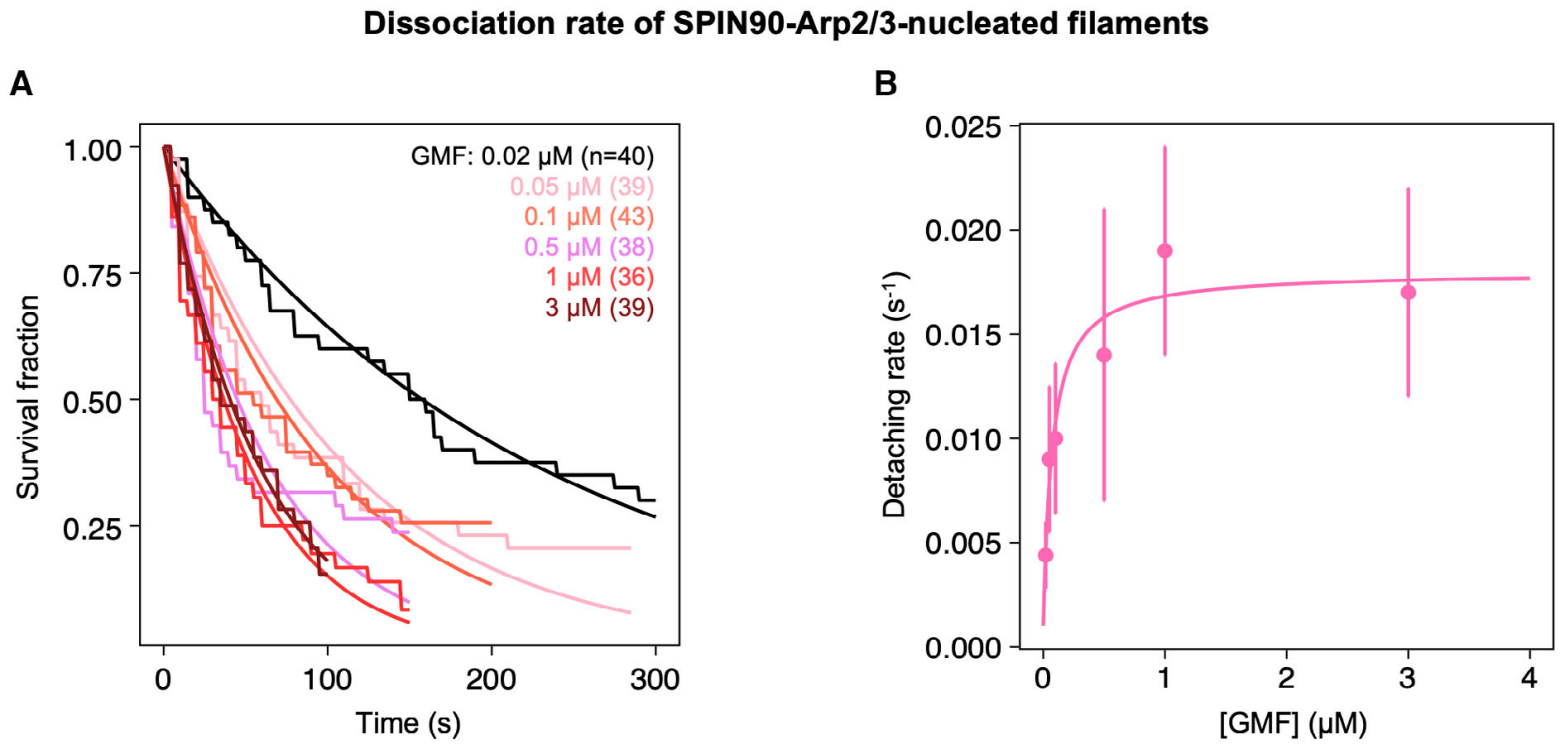
GMF accelerates the detachment of SPIN90‐Arp2/3‐nucleated actin filaments The fraction of filaments still attached to the surface by SPIN90‐Arp2/3, over time, when exposed to different concentrations of GMF. For each experiment, *n* = 36–43 filaments. Black lines are exponential fits. Each experiment was repeated three times (technical replicates), yielding similar results.Detachment rates, determined by exponential fits of survival curves shown in (A), as a function of the concentration of GMF. The error bars are determined by fitting the edges of the 95% confidence intervals (not shown, for readability) of the survival curves. The data are fitted by a Michaelis–Menten equation, yielding an apparent dissociation constant K_D_ = 70 ± 23 nM and a maximum detachment rate of 0.017 ± 0.001 s^−1^. Source data are available online for this figure.

Given our observations, we wondered whether branch destabilizers such as GMF also impact the stability of SPIN90‐Arp2/3‐nucleated filaments. To do so, we performed detachment experiments similar to the ones in Fig 2A, where we exposed the filaments to different concentrations of GMF. We found that GMF accelerated the detachment of filaments from the surface (Fig 3A and B), with a higher affinity than VCA (Fig 2A–C): K_D_ = 70 ± 23 nM for GMF compared to 100–820 nM for the VCA motifs from different NPFs. The maximum dissociation rate, obtained with saturating amounts of GMF (plateau value of Michaelis–Menten fit), was 0.016 ± 0.002 s^−1^ (average and standard deviation from three independent sets of data, similar to Fig 3B).

### VCA and GMF generate free pointed ends as they dissociate Arp2/3 from both SPIN90 and the filament

So far, our readout of the destabilization of SPIN90‐Arp2/3 at the filament pointed end is the detachment of the filament from the surface. Since SPIN90 is strongly anchored to the surface via a His‐tag (see Materials and Methods), the detachment of the filament could result either from the rupture of the SPIN90‐Arp2/3 bond or from the removal of the Arp2/3 complex from the filament‐pointed end.

**Figure 4:**
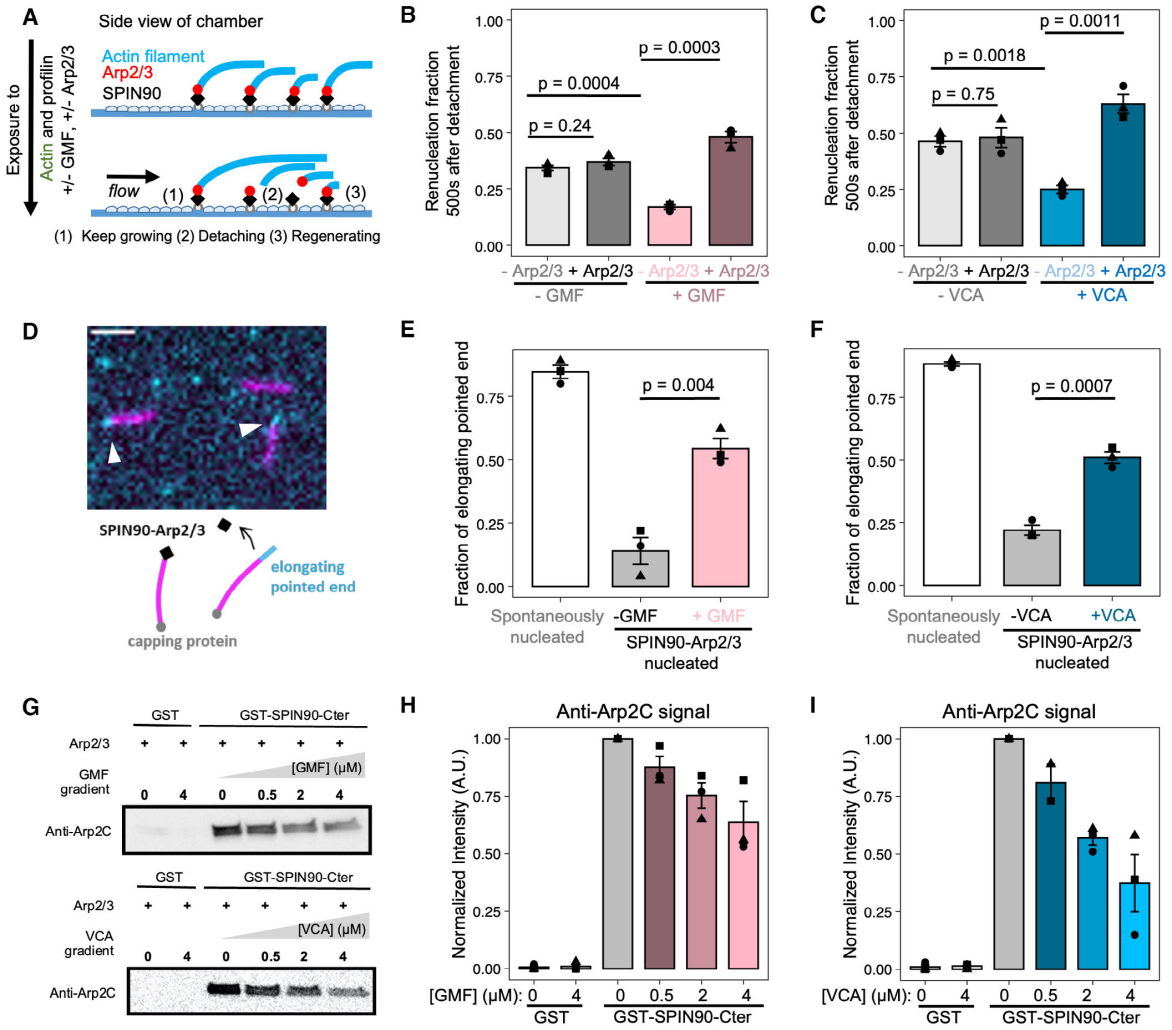
GMF and VCA accelerate the detachment of Arp2/3 from both SPIN90 and filament pointed ends A–CAssessing the presence of Arp2/3 on SPIN90 after filament departure. (A) Schematic of the renucleation assay, using microfluidics. The SPIN90‐Arp2/3 complex‐decorated surface is exposed to 2 μΜ Alexa488 (15%)‐G‐actin and 1 μΜ profilin, with 0 or 0.5 μΜ GMF, and with 0 or 5 nM Arp2/3. Filaments nucleated by SPIN90‐Arp2/3 elongate (1) and, over time, detach from the surface (2). Some of them re‐nucleate, from the same location (3). (B, C) The fraction of SPIN90 that re‐nucleated a filament 500 s after the detachment of the initial filament was quantified for different conditions.D–FAssessing the presence of Arp2/3 at the filament pointed end. (D) Schematic and microscope image of the pointed end uncapping assay. Preformed filaments made with Alexa488 (15%)‐actin (magenta) were mixed with 10 nM Capping protein, 1 μΜ Alexa568 (15%)‐G‐actin (cyan), and 0 or 0.5 μΜ GMF (or 0.5 μM VCA). In the microscope image, filaments were nucleated by SPIN90‐Arp2/3, and exposed to 0.5 μM GMF. Slow elongation of the filaments at one end (white arrowheads) indicates freely growing pointed ends. Scale bar: 3 μm. (E, F) The fraction of elongating, that is, not capped, pointed ends was determined for different conditions.G–IGMF and VCA interfere with the SPIN90‐Arp2/3 interaction in the absence of filament. (G) Immunoblots from pull‐down assays where GST beads, decorated with GST or GST‐SPIN90‐Cter (the functional domain of SPIN90), were incubated with 0.4 μΜ Arp2/3 and gradient concentrations of GMF or VCA for 1 h at room temperature. After the unbound protein was washed out, the amount of Arp2/3 attached to the beads was detected by anti‐ArpC2 antibody. Ponceau red straining of the membrane verified that the beads were loaded with equal amounts of GST or GST‐SPIN90 (Appendix Fig S2). (H, I) Quantification of the amount of ArpC2 remaining on the beads in different conditions. Data information: In all graphs (B, C, E, F, H and I): the bars indicate the average values, and the error bars indicate the standard deviations, from three independent repeats of each experiment (data points). For each condition (in B, C, E and F), a total of *n* = 70–152 filaments were analyzed (see Source Data for individual values of *n*). The *P*‐values (in B, C, E and F) were determined with an unpaired *t*‐test. Source data are available online for this figure.

To determine whether the SPIN90‐Arp2/3 interaction is destabilized by GMF and VCA, we examined whether the Arp2/3 complex was still bound to SPIN90 after the detachment of the actin filament, in our microfluidics assay (Fig 4A–C). To do so, we quantified the fraction of functional SPIN90 locations that were able to re‐nucleate a filament, following the departure of the initial actin filament, in different conditions. The new filaments took several tens of seconds to appear, consistent with re‐nucleation, and ruling out regrowth from broken filaments which would be instantaneous. Based on the density of nucleated filaments in our experiments, it is very unlikely that re‐nucleated filaments result from a different, co‐localized SPIN90.

When only actin and profilin were flowed into the chamber, we observed that 36% of surface‐anchored SPIN90 were able to re‐nucleate a filament, within the 500 s following the detachment of their first filament (Fig 4A–C). Based on our earlier experiments (Fig 1B), this number is the expected nucleation fraction after that time. This confirms that the population of anchored SPIN90 from which filaments were first nucleated is still attached to the surface. This also indicates that, upon detachment of the filament, the Arp2/3 complex stays bound to SPIN90. Consistent with this interpretation, supplying fresh Arp2/3 during the experiment did not increase the fraction of re‐nucleated filaments (Fig 4B and C).

In the presence of GMF, however, the re‐nucleation fraction dropped to 15%, and was rescued when fresh Arp2/3 was supplied (Fig 4B). This indicates that GMF destabilizes the SPIN90‐Arp2/3 bond in the presence of actin. We obtained similar results with the VCA motif of N‐WASP (from here onward, VCA refers to GST‐NWASP‐VCA), indicating that VCA also destabilizes the SPIN90‐Arp2/3 bond in the presence of actin (Fig 4C). However, these experiments do not tell us if the separation of Arp2/3 from SPIN90 causes filament detachment, or if it occurred after. They also do not indicate whether Arp2/3 remains attached to the pointed end of the departing filament.

To determine whether the Arp2/3 complex remained bound to the pointed ends of the filaments exposed to GMF or VCA, we performed experiments in open chambers (no microfluidic flow) where we could monitor the filaments' ability to elongate from their pointed ends. To evaluate the fraction of capped pointed ends, we exposed pre‐formed Alexa488 (15%)‐actin filaments to barbed end capping proteins and to Alexa568 (15%)‐G‐actin (Fig 4D). The vast majority of spontaneously nucleated filaments were able to elongate from their pointed ends, validating the rationale of the assay (Fig 4E and F). In contrast, most filaments nucleated by SPIN90‐Arp2/3 were unable to do so, indicating that their pointed ends were capped by SPIN90‐Arp2/3. In the presence of GMF or VCA, the fraction of elongating pointed ends increased significantly (Fig 4E and F). This demonstrates that the Arp2/3 complex was removed from the pointed end following, or during, the destabilization of SPIN90‐Arp2/3 by GMF or VCA.

To determine if SPIN90‐Arp2/3 is also destabilized by GMF or VCA in the absence of a filament, we performed bead pull‐down assays. We show that GMF and VCA both decrease the amount of Arp2/3 bound to SPIN90‐decorated beads (Fig 4G–I). This observation indicates that, in the absence of actin, GMF and VCA interfere with the binding of Arp2/3 to SPIN90.

### Piconewton forces promote debranching, but have no impact on SPIN90‐nucleated filaments

**Figure 5:**
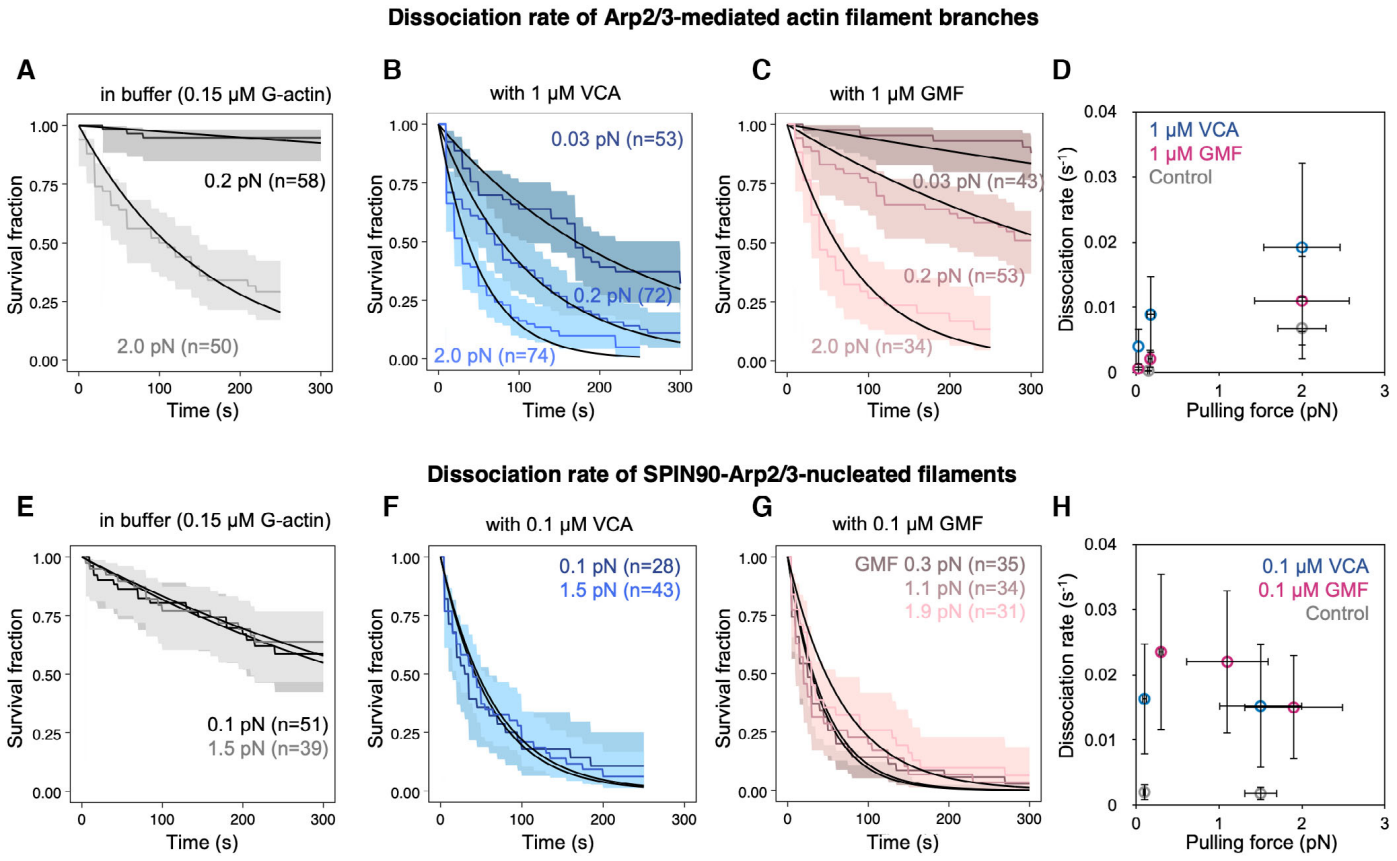
Impact of mechanical tension on debranching and on the detachment of SPIN90‐Arp2/3‐nucleated filaments A–CSurvival fractions for branches exposed to different forces in the microfluidics chamber, while being exposed to 0.15 μM G‐actin, alone (gray curves, A), with 1 μM VCA (blue curves, B), or with 1 μM GMF (red curves, C). The shaded areas represent 95% confidence intervals. Black lines are exponential fits.DSummary of the debranching rates, resulting from the exponential fits in (A–C).E–GSurvival fractions for SPIN90‐Arp2/3‐nucleated filaments exposed to different forces in the microfluidics chamber, while being exposed to 0.15 μM G‐actin, alone (gray curves, E), with 0.1 μM VCA (blue curves, F), or with 0.1 μM GMF (red curves, G). The shaded areas represent 95% confidence intervals. Black lines are exponential fits.HSummary of the detachment rates for SPIN90‐Arp2/3‐nucleated filaments, resulting from the exponential fits in (E–G). Data information: Each curve in (A–C, E–G) is from a single experiment. Each experiment was repeated twice (technical replicates), yielding similar results. The results in (E, F) were confirmed by additional experiments on linear filaments, with a different anchoring strategy for SPIN90 and with a higher VCA concentration (Fig EV4). In (D, H), horizontal error bars are SD of applied forces, and vertical error bars result from fits of the 95% confidence interval in the survival fractions. Source data are available online for this figure.

It was recently shown that debranching is accelerated when (sub) piconewton pulling forces are applied to the branch, regardless of the orientation of the force (Pandit *et al*, 2020). We, therefore, wondered whether tensile forces also destabilize SPIN90‐Arp2/3 at the pointed end of filaments. By controlling the flow rate in our microfluidics assay, we can vary the force applied to actin filaments (Jégou *et al*, 2013), in a range similar to what has been measured on individual filaments and NPFs in cells (Jiang *et al*, 2003; Mehidi *et al*, 2021). Forces ranging from 0.2 to 2 pN accelerated debranching (Fig 5A). This observation remained true in the presence of VCA and GMF: pulling forces further accelerated debranching (Fig 5B–D). In contrast, we found that forces up to 2 pN had no impact on the detachment of SPIN90‐Arp2/3‐nucleated filaments, even in the presence of VCA or GMF (Fig 5E–H).

**Figure EV4:**
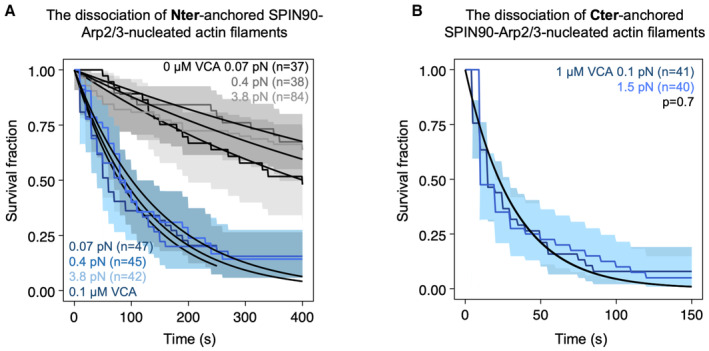
Anchoring SPIN90 by its C‐ or N‐terminus has no impact on its behavior Survival fractions for SPIN90‐Arp2/3‐nucleated filaments exposed to different forces in the microfluidics chamber, while being exposed to 0.15 μM G‐actin alone or with 0.1 μΜ GFP‐VCA. In this case, SPIN90 was anchored on the surface through its Nter‐GST‐tag. Black lines are exponential fits, yielding dissociation rates at 0.07 pN force, koff = (1.6 ± 0.6) × 10^−3^ s^−1^ without VCA, and koff = (7.5 ± 3.3) × 10^−3^ s^−1^ with 0.1 μΜ VCA; at 0.4 pN force, koff = (1.2 ± 0.5) × 10^−3^ s^−1^ without VCA, and koff = (7.4 ± 3.5) × 10^−3^ s^−1^ with 0.1 μΜ VCA; 3.8 pN, koff = (1.3 ± 0.4) × 10^−3^ s^−1^ without VCA, and koff = (6.9 ± 2.4) × 10^−3^ s^−1^ with 0.1 μΜ VCA. The shaded areas represent 95% confidence intervals.Survival fractions for SPIN90‐Arp2/3‐nucleated filaments exposed to 1 μM VCA. In this case, SPIN90 was anchored on the surface through its Cter‐His‐tag. Black lines are exponential fits, yielding dissociation rates koff = (3.1 ± 1.5) × 10^−2^ s^−1^ at 0.1 pN force and koff = (3.1 ± 1.3) × 10^−2^ s^−1^ at 1.5 pN force. The shaded areas represent 95% confidence intervals.

To make sure that this result does not depend on our anchoring SPIN90 by its C‐terminus (see Materials and Methods), we also attached the protein by its N‐terminus (Fig EV4A). Again, we found that mechanical tension (up to 3.8 pN) had no impact on filament detachment. This suggests that the SPIN90‐Arp2/3 interaction can resist to a piconewton pulling force regardless of its orientation. We also verified that tension had no impact on detachment when exposing the filaments to GFP‐VCA instead of GST‐VCA (Fig EV4A), or to a higher concentration (1 μM) of GST‐VCA (Fig EV4B).

**Figure 6:**
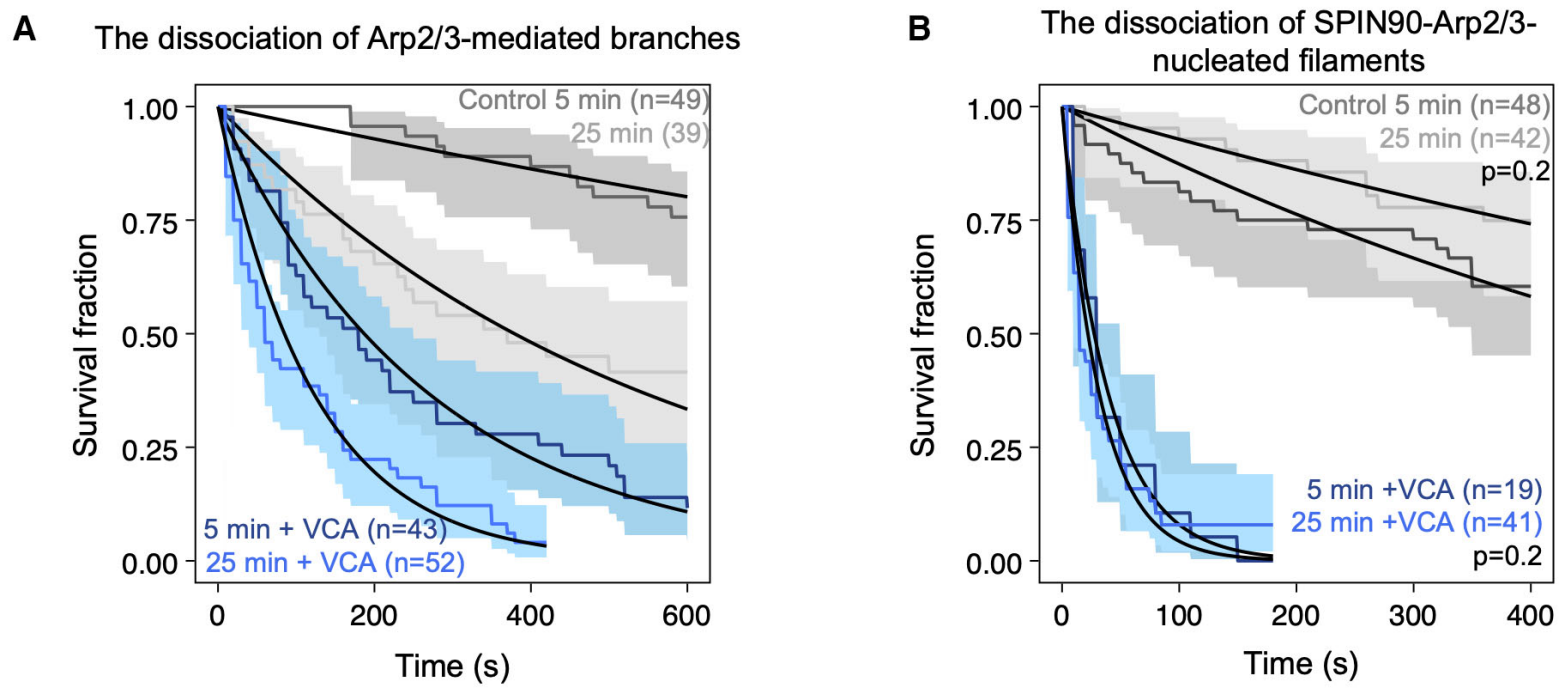
Aging destabilizes branches but has no measurable effect on SPIN90‐Arp2/3‐nucleated filaments Fraction of remaining branches, over time, starting at an average of 5 and 25 min after branch nucleation, in the presence (blue) or absence (gray) of VCA. Lines are exponential fits, yielding debranching rates of (3.7 ± 0.6) × 10^−4^ s^−1^ and (20 ± 8) × 10^−4^ s^−1^ without VCA for 5‐ and 25‐min‐old branches, respectively; and debranching rates of (3.8 ± 1.3) × 10^−3^ s^−1^ and (8.2 ± 2.8) × 10^−3^ s^−1^ with 0.5 μM VCA for 5‐ and 25‐min‐old branches, respectively.Fraction of attached SPIN90‐nucleated filaments, over time, starting at an average of 5 and 25 min after branch nucleation, in the presence (blue) or absence (gray) of VCA. Lines are exponential fits, yielding detachment rates of (12 ± 7) × 10^−4^ s^−1^ and (7 ± 3) × 10^−4^ s^−1^ without VCA for 5‐ and 25‐min‐old filaments, respectively (*P* = 0.2, log‐rank test); and detachment rates of (2.5 ± 1.1) × 10^−2^ s^−1^ and (3.1 ± 1.7) × 10^−2^ s^−1^ with 1 μM VCA for 5‐ and 25‐min‐old filaments, respectively (*P* = 0.4, log‐rank test). Data information: Shaded areas represent 95% confidence intervals. Each curve is the result of a single experiment. The experiments on the aging of SPIN90‐Arp2/3 (B) were repeated twice, yielding the same result. The flowing solutions applied a weak force to the filaments (< 1 pN) and to the branches (0.2 pN). Source data are available online for this figure.

### Aging promotes debranching but has no impact on SPIN90‐nucleated filaments

Debranching is enhanced as ATP is hydrolyzed in Arp2 and Arp3, within the Arp2/3 complex at a branch junction, within minutes of “daughter” filament nucleation (Le Clainche *et al*, 2003; Pandit *et al*, 2020). Branches thus become less stable over time. Quantifying ATP hydrolysis in SPIN90‐activated Arp2/3 is particularly challenging because of the large background of ATP hydrolysis taking place in F‐actin, and because SPIN90 activates even lower amounts of Arp2/3 than the branching reaction. To gain insights into this question, we therefore sought to determine if time destabilizes SPIN90‐Arp2/3 at filament pointed ends, as seen with branches.

In our experiments, up to now, we observed the detachment of branches and SPIN90‐nucleated filaments on average 5 min following their nucleation. Here, we aged filaments and branches for an additional delay while exposing them to minimal forces with a gentle flow, and subsequently, monitored their detachment, on average 25 min after their nucleation.

We confirmed that 25‐min‐old branches detach faster than 5‐min‐old branches, and found that this remains true when debranching is enhanced by VCA (Fig 6A). In contrast, we observed no significant difference between 5‐ and 25‐min‐old SPIN90‐Arp2/3‐nucleated filaments, with or without VCA (Fig 6B).

### Cortactin stabilizes branches and SPIN90‐Arp2/3 at filament pointed ends

**Figure 7:**
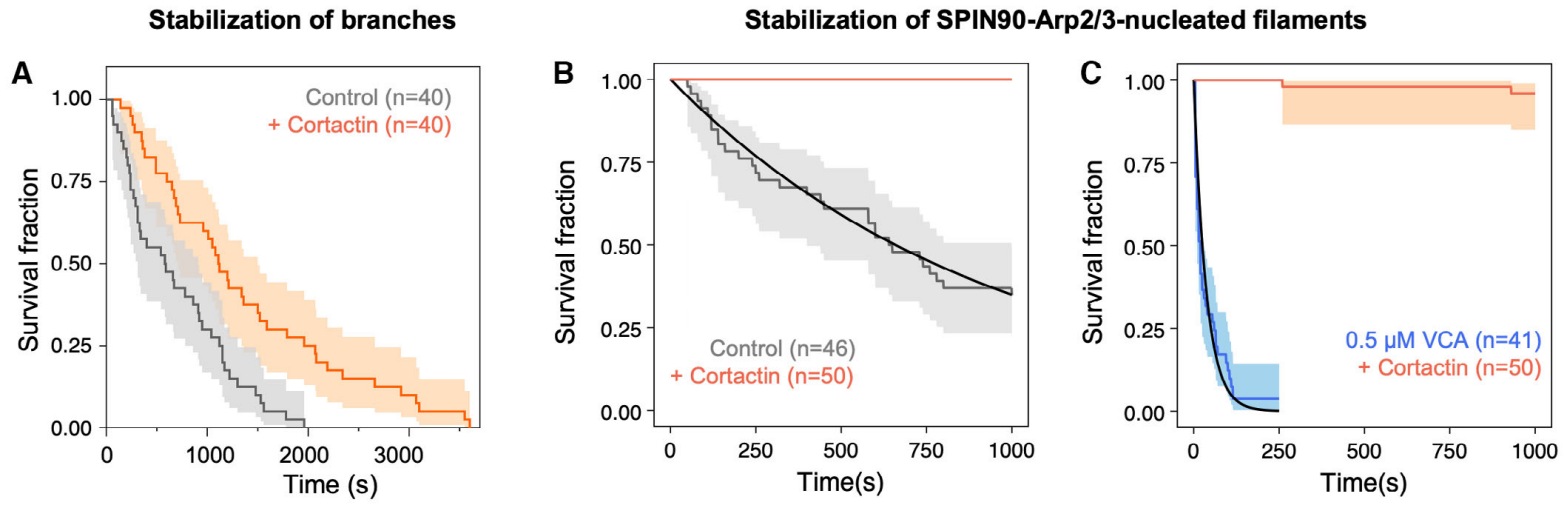
Cortactin stabilizes Arp2/3‐mediated branches as well as SPIN90‐Arp2/3 at the pointed end of filaments The fraction of remaining branches, versus time, exposed to 0.16 μM G‐actin (black) supplemented with 50 nM cortactin (red). The branch junctions were exposed to an average force of 1.4 pN. Similar results were obtained with different forces and different protein concentrations (Appendix Fig S3).The fraction of remaining SPIN90‐Arp2/3‐nucleated filaments, versus time, exposed to 0.15 μM G‐actin (black) supplemented with 10 nM cortactin (red).The fraction of remaining SPIN90‐Arp2/3‐nucleated filaments, versus time, exposed to 0.15 μM G‐actin, with 0.5 μM VCA (black) supplemented with 10 nM cortactin (red). Data information: Shaded areas represent 95% confidence intervals. Each curve is the result of a single experiment. Repeats of experiments in (A) with different forces and aging are shown in Appendix Fig S3. The experiments shown in (B, C) were repeated three times, yielding similar results. Source data are available online for this figure.

Finally, we asked if cortactin, a protein which interacts with the Arp2/3 complex to stabilize branches (Weaver *et al*, 2001; Abella *et al*, 2016), could have a similar effect on SPIN90‐Arp2/3‐nucleated filaments. Since the stabilizing activity of cortactin was never observed directly on filament branches, we sought to confirm this property by carrying out debranching experiments as in Fig 2D. We observed that 30–100 nM cortactin indeed slows down debranching, by approximately twofold (Fig 7A and Appendix Fig S3).

We next monitored the nucleation and the detachment of actin filaments from a SPIN90‐Arp2/3‐decorated surface, in the presence of cortactin. We found that cortactin very efficiently stabilizes SPIN90–Arp2/3 at filament pointed ends (Fig 7B): 10 nM cortactin were enough to prevent all filament dissociation from the surface over the course of the experiment (1,000 s). We then wondered what would be the outcome of SPIN90‐nucleated filaments in the presence of both cortactin and a destabilizer like VCA. We found that, in the presence of 0.5 μM VCA, the addition of 10 nM cortactin reduces the filament detachment rate by 3 orders of magnitude (Fig 7C).

**Figure EV5:**
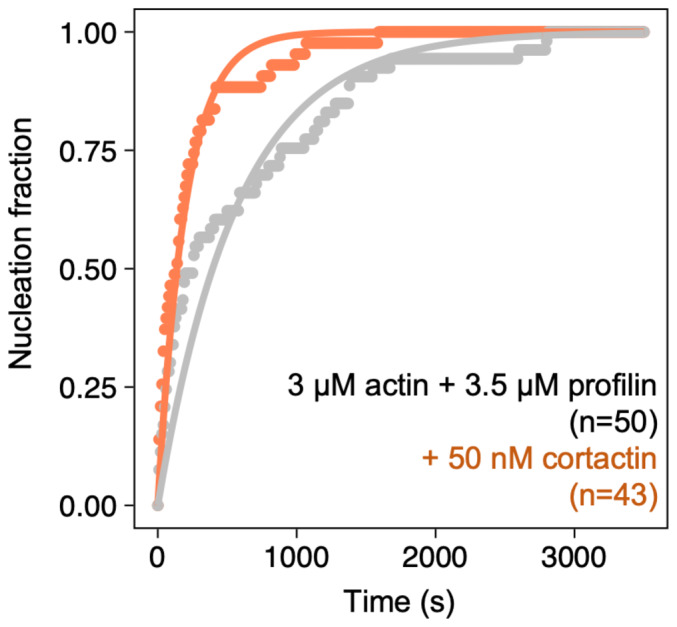
SPIN90‐Arp2/3 filament nucleation is accelerated by cortactin Normalized number of filaments nucleated over time, from SPIN90‐Arp2/3 exposed to 3 μΜ G‐actin (15% labeled with Alexa488) and 3.5 μΜ profilin, with 0 or 50 nM of cortactin. Solid lines are exponential fits, yielding nucleation rates knuc = (2.0 ± 0.04) × 10^−3^ s^−1^ without cortactin, and knuc = (5.3 ± 0.08) × 10^−3^ s^−1^ with cortactin. Indicated values of n are the number of filaments observed in each experiment.

We also observed that cortactin accelerates the nucleation of filaments by SPIN90‐Arp2/3, even in the presence of an excess of profilin (Fig EV5). The latter result is in contrast with VCA, whose enhancement of SPIN90‐induced nucleation requires G‐actin (Fig 1). This observation is consistent with the fact that cortactin does not bind G‐actin.

## Discussion

In cells, the Arp2/3 complex can be activated by VCA to generate branches, or by SPIN90 to generate linear filaments. Whether these two mechanisms can be regulated separately is an outstanding question. Here, we measured the impact of different regulatory factors on both mechanisms.

We found that both branched and linear Arp2/3‐generated filaments were stabilized by cortactin, and destabilized by GMF as well as by the VCA motifs of NPFs. While these regulatory proteins similarly affect both systems, they do so with clear quantitative differences. For example, SPIN90‐Arp2/3 at pointed ends is destabilized by lower concentrations of GMF, compared to branches (Fig 5). Also, cortactin appears far more efficient at stabilizing SPIN90‐Arp2/3 at the pointed end than at stabilizing branches (Fig 7). This result was unexpected, considering that cortactin is proposed to stabilize branch junctions by simultaneously binding to the Arp2/3 complex and to the mother filament (Helgeson & Nolen, 2013). It indicates that, in the absence of a mother filament, cortactin can simultaneously bind to the Arp2/3 complex and to the Arp2/3‐nucleated filament, and raises the possibility that the same could also happen when cortactin stabilizes a branch. In the absence of direct structural data of cortactin bound to branch junctions, this is an open question. Our observation that cortactin accelerates the activation of Arp2/3 by SPIN90 (Fig EV5), here again in the absence of a mother filament, further highlights that our understanding of cortactin is incomplete.

In addition, we found that VCAs from different NPFs did not rank the same in their ability to destabilize SPIN90‐Arp2/3 and branches (Figs 2 and EV1). Recent cryo‐EM data reveal small differences in the movement of Arp2 and in the flattening of Arp3, upon activation of the Arp2/3 complex in the two situations (Ding *et al*, 2022). These differences possibly affect their interaction with the C‐ and A‐domains of the NPFs. The strong sequence similarities between the C‐ and A‐domains of different NPFs suggest that their linker regions may also play a role (Appendix Fig S4). For instance, the long linkers of WASH, between its V‐ and C‐domain as well as between its C‐ and A‐domain, may ease its interaction with the Arp2/3 complex, and partly explain why WASH is the most efficient of our three NPFs at enhancing debranching. Comparing WASP and NWASP, the longer and highly negatively charged linker between the C‐ and A‐domain of NWASP may interact more easily with two positive charged regions of Arp2 (K299 and K341) and ArpC1 (R94, R97, K138, and K138; Fäßler *et al*, 2020; Zimmet *et al*, 2020). This may explain in part why NWASP is more efficient than WASP at destabilizing both SPIN90‐Arp2/3 and branch junctions. In addition, we report here that the destabilization of SPIN90‐Arp2/3 at pointed ends is solely due to the C‐ and A‐domains, while the enhancement of debranching also involves the V‐domain (Fig EV3). NWASP having two V‐domains, while WASP and WASH only have one (Appendix Fig S4), may therefore enhance its ability to destabilize branches but not SPIN90‐Arp2/3 at pointed ends.

In any case, the quantitative biochemical differences we report for the action of VCA, GMF, and cortactin on branched versus linear Arp2/3‐generated filaments suggest that proteins in cells may differently regulate these two types of filaments. Regulatory proteins could thus provide a means to control the two Arp2/3 machineries independently.

Additional factors may further tune these regulatory activities. For instance, we found that aging and mechanical forces, which both strongly favor debranching, have little effect on the stability of SPIN90‐Arp2/3 at pointed ends (Figs 5 and 6). This is an important feature in the cellular context, where actin networks containing both types of Arp2/3‐generated filaments, such as the cell cortex or during endocytosis, are exposed to mechanical stress. It indicates that mechanical stress can contribute to debranching, but that regulatory proteins are likely required to remove SPIN90 from the pointed ends.

The differences we observe, where it appears that SPIN90‐Arp2/3 is globally more sensitive to regulatory proteins and less sensitive to aging and mechanical tension than branch junctions, can also be linked to conformational differences between activated Arp2/3 in the two situations. Branch junctions become less stable as ATP in Arp2 and Arp3 is hydrolyzed, within minutes after a branch is formed (Le Clainche *et al*, 2003; Dayel & Mullins, 2004). In contrast, ATP is still detected in Arp2 and Arp3 by cryo‐EM, hours after activation by SPIN90 (Shaaban *et al*, 2020). Consistently, compared to Arp3 in SPIN90‐activated Arp2/3, Arp3 in branch junctions adopts a flatter conformation, associated with the triggering of ATP hydrolysis (Ding *et al*, 2022). This suggests that SPIN90‐activated Arp2/3 does not hydrolyze ATP, or does so very slowly, and is consistent with our observation that SPIN90‐Arp2/3 does not seem to age (Fig 6).

The structural basis for the mechanical robustness of SPIN90‐Arp2/3 is less clear. SPIN90 binds primarily to ArpC4, while, in a branch junction, the mother filament forms an extensive interface with several subunits of Arp2/3 (Luan *et al*, 2018; Fäßler *et al*, 2020; Ding *et al*, 2022). One could thus have expected Arp2/3 to detach more readily from SPIN90 than from the mother filament. A possibility would be that, in spite of very similar conformations for the two active states of Arp2/3, differences arise in the conformational transitions between intermediate states upon the application of a mechanical load, similar to what has recently been proposed for the different nucleotide states of F‐actin (Reynolds *et al*, 2022).

Our observation that VCA enhances debranching (Fig 2) indicates that VCA can bind to Arp2/3 at the branch junction. This was unexpected because VCA detaches from Arp2/3 on the side of the mother filament during the branching reaction (Smith *et al*, 2013). This result further suggests that VCA, beyond its role in the formation of branched filament networks, may also contribute to their reorganization by enhancing debranching. In cells, NPFs expose their VCA motifs upon activation and binding to the membrane, meaning that branch junctions would need to contact the NPF‐decorated membrane in order for debranching to be enhanced. This situation could be encountered during endocytosis, for example, where the branched filament network is densely packed against the membrane, and where debranching appears to be required to complete endocytic internalization (Martin *et al*, 2006). Similarly, the NPF WAVE has been observed within the lamellipodium of migrating cells, likely on the dorsal and ventral membranes, in addition to its localization at the leading edge where the branching reaction occurs (Mehidi *et al*, 2021; Kage *et al*, 2022). Branch junctions within the lamellipodium could thus encounter membrane‐bound WAVE. A limitation to VCA‐induced debranching, however, would come from the high concentration of cytoplasmic profilin‐actin, which would bind to the polyproline domain of NPFs and ultimately load G‐actin onto their V‐domain (Bieling *et al*, 2018). Unless G‐actin is locally depleted, this would likely inhibit, at least partially, the destabilizing activity of NPFs (Fig EV3).

In cells, where the turnover of actin networks is tightly controlled, the state of filament pointed ends is of primary importance. Early EM observations of branched filaments detected the Arp2/3 complex also at the pointed end of linear filaments, suggesting that Arp2/3 could act as a pointed end capper (Mullins *et al*, 1998). The persistence of SPIN90 with Arp2/3 at pointed ends makes this cap very stable, blocking filament depolymerization and re‐annealing, and preventing SPIN90 from nucleating other filaments (Balzer *et al*, 2019). We show that the destabilization of SPIN90‐Arp2/3 by GMF or VCA results in free pointed ends and renews the pool of free SPIN90 and Arp2/3 complexes (Fig 4). In cells, we expect such mechanisms to contribute to controlling the state of pointed ends, and thus to controlling the stability and turnover of actin filament networks. Future studies should reveal if other inhibitors of branching, like Arpin or coronin, can play a similar role with SPIN90‐Arp2/3 at pointed ends.

## Materials and Methods

### Biochemistry

#### Protein preparation

Skeletal muscle actin was purified from rabbit muscle acetone powder following the protocol from (Spudich & Watt, 1971), as described in Wioland *et al* (2017).

We have recombinantly expressed the following proteins in bacteria, with a N‐terminal GST tag: human N‐WASP‐VCA (with its two V‐domains) and N‐WASP‐CA (392–505 and 453–505, respectively, uniprot O00401), human WASP‐VCA (418–502, uniprot P42768), mouse WASH‐VCA (319–475), human SPIN90 full‐length (1–715, uniprot Q9NZQ3–3), SPIN90 Cter (267–715), and mouse cortactin full length (1–546 Q60598). Expression was performed in *Escherichia coli* Rosetta 2 (DE3) for 16 h at 18°C. GST‐tagged proteins were purified by affinity chromatography over a Sepharose 4B GSH affinity column (Cytiva) in GST buffer (20 mM Tris pH 7.5, 500 mM NaCl, and 1 mM DTT). The GST protein was eluted with the same buffer supplemented with 50 mM GST. The purification is followed by gel filtration over a Hiload Superdex 200 column (Cytiva) in GF‐buffer (20 mM Tris pH 7.5, 50 mM KCl, 1 mM DTT, and 5% glycerol). Before the gel filtration step, the GST‐tag of GFP‐N‐WASP‐VCA and cortactin was cut by PreScission Protease (Sigma‐Aldrich) overnight at 4°C to obtain monomeric N‐WASP‐VCA and untagged full‐length cortactin.

C‐terminal His‐tagged versions of recombinant human SPIN90 full‐length (1–722) and drosophila GMF (1–138, uniprot Q9VJL6, plasmid given by the Lappalainen lab (Poukkula *et al*, 2014)) were purified by affinity chromatography over a HisTrap HP column (Cytiva) in His‐buffer (20 mM Tris pH 7.5, 500 mM NaCl, 1 mM DTT, and 20 mM imidazole) and eluted in the same buffer supplemented with 300 mM imidazole. The purification was polished by gel filtration over a Hiload Superdex 200 column (Cytiva) in 20 mM Tris pH 7.5, 500 mM NaCl, and 1 mM DTT.

Recombinant human profilin1 was expressed and purified, as described in Cao *et al* (2018).

Arp2/3 was purified from bovine brain, as previously described (Le Clainche & Carlier, 2004).

#### Protein labeling

Actin was fluorescently labeled on the surface lysine 328, using Alexa‐488 or Alexa‐594 succinimidyl ester (Life Technologies), as described in detail in Romet‐Lemonne *et al* (2018). To minimize potential artifacts caused by the presence of the fluorophores, we typically used an actin labeling fraction of 15%.

### Microscopy

#### Buffers

All microfluidics experiments were performed in F‐buffer: 5 mM Tris–HCl pH 7.0, 50 mM KCl, 1 mM MgCl_2_, 0.2 mM EGTA, 0.2 mM ATP, 10 mM DTT, 1 mM DABCO, and 0.1% BSA. Open chambers experiments (Fig 4D–F) were performed using the same buffer supplemented with 0.3% methylcellulose 4,000 cP.

#### Single‐filament assays in microfluidics

Microfluidics experiments were done with Poly‐Dimethyl‐Siloxane (PDMS, Sylgard) chambers based on the original protocol from Jégou *et al* (2011). A step‐by‐step description of this protocol can be found in Wioland *et al* (2022) doi:10.3791/63891. The chambers have cross‐shaped channels with three inlets and one outlet. A MFCS system and Flow Units (Fluigent) were used to control and monitor the microfluidic flows. PDMS chambers were cleaned and mounted by following the protocol described in Cao *et al* (2018). The experiments were performed on a Nikon TiE inverted microscope, equipped with a 60× oil‐immersion objective, in F‐buffer. The temperature was controlled and set to 25°C (using an objective heater from Oko‐lab). Actin filaments were visualized using TIRF microscopy (iLAS2, Gataca Systems) with 150 mW tunable lasers. Images were acquired using an Evolve EMCCD camera (Photometrics), controlled with the Metamorph software (version 7.10.4, from Molecular Devices).

#### SPIN90‐Arp2/3 assays

Full‐length GST‐SPIN90 or SPIN90‐His was specifically anchored to the coverslip surface of a microfluidics chamber using specific antibodies against GST (Rockland, 600‐106‐200) or against 6xHis (Qiagen, 34440), adsorbed to the surface prior to passivation by exposing the chamber to a 3% BSA solution for at least 5 min, as described in Cao *et al* (2020). The chamber was subsequently exposed to 30 nM Arp2/3 complex for 5 min and rinsed with F‐buffer.

##### SPIN90‐Arp2/3 filament nucleation

The nucleation rate is measured by exposing surface‐anchored His‐tagged SPIN90‐Arp2/3 to 2 μM 15% Alexa‐488 G‐actin and 1 μM profilin, with or without 0.5 μM of the different VCA motifs (Fig 1), or in the presence or absence of 50 nM cortactin (Fig EV5). In these conditions, there is no spontaneous nucleation: no filaments were detected growing from the surface in control experiments performed without SPIN90 or without Arp2/3.

##### Dissociation of SPIN90‐Arp2/3‐nucleated filaments

Surface‐anchored SPIN90‐Arp2/3 was exposed to 0.7 μM 15% Alexa‐488 actin for 5 min to nucleate and elongate actin filaments. The filaments were then exposed to 0.15 μM G‐actin, in order to maintain their length constant, and to different concentrations of regulatory proteins, and the detachment of the filaments was monitored. The survival fractions of SPIN90‐Arp2/3‐nucleated filaments over time were fitted with a single exponential function that yields the dissociation rates of filament from SPIN90‐Arp2/3 for each condition.

Different concentrations of different VCA motifs were used (Fig 2A–C), as well as 1 μM NWASP‐CA (Fig EV3C) and 0.2 μΜ N‐WASP‐VCA and various concentrations of G‐actin (Fig EV3A). The dissociation rates were plotted versus the concentration of VCA, and a fit using the Michaelis–Menten equation was used to obtain the affinity constant and maximum dissociation rate (Fig 2C).

The dissociation rate of SPIN90‐Arp2/3‐nucleated actin in the presence of cortactin was measured following the same procedure (Fig 7).

The dissociation rate of SPIN90‐Arp2/3‐nucleated actin in the presence of GMF was measured following the same procedure but using the full‐length GST‐SPIN90 construct (Fig 3).

To measure the impact of aging on the dissociation rate, generated actin filaments were incubated with 0.15 μM G‐actin for an extra duration of 20 min, before being exposed to buffer only or 0.5 μM of N‐WASP‐VCA (Fig 6).

##### Impact of mechanical tension on the detachment of SPIN90‐Arp2/3‐nucleated filaments

With the same experimental procedure described in the previous paragraph, various pulling forces can be applied to the filaments by varying the flow rate and filament length (Jégou *et al*, 2013; Fig 5).

##### Detachment of Arp2/3 from both SPIN90 and the filament pointed end (Fig 4)

To assess the ability of GMF to separate Arp2/3 from SPIN90, surface‐anchored GST‐SPIN90‐Arp2/3‐nucleated filaments were exposed to 2 μM 15% Alexa‐488‐labeled G‐actin, 1 μM profilin, with and without 0.5 μM GMF, and with or without 5 nM Arp2/3 (Fig 4B). The renucleation fraction of a randomly picked population of SPIN90‐Arp2/3‐nucleated filaments was assessed after 500 s.

To assess the ability of GST‐N‐WASP‐VCA to separate Arp2/3 from SPIN90, surface‐anchored SPIN90‐His‐Arp2/3‐nucleated filaments were alternatively exposed to 0.5 μM GST‐N‐WASP‐VCA or buffer for 30 s, then to a nucleation solution containing 2 μM 15% Alexa‐488‐labeled actin and 1 μM profilin, with or without 5 nM Arp2/3 for 90 s. In this assay, VCA and Arp2/3 were not mixed together with actin to avoid unnecessary generation of branched actin filaments in solution and on the surface‐anchored filaments. The renucleation fraction of a randomly picked population of SPIN90‐Arp2/3‐nucleated filaments was assessed after 500 s (Fig 4C).

##### Single filaments in open chambers (Fig 4D–F)

Experiments aimed at observing pointed end elongation were not performed using microfluidics, but using so‐called “open chambers,” that is, mPEG‐silane‐passivated flow chambers made following the protocol described in Cao *et al* (2020). Fifty nanomolar Arp2/3, 1 μM SPIN90 Cter, and 0.5 μM 15% Alexa‐488 actin were mixed together for 3 min to generate SPIN90‐Arp2/3‐nucleated actin filaments. As a positive control, 1 μM 15% Alexa‐488 actin were mixed together for 3 min to generate spontaneously nucleated actin filaments. Four microliter of SPIN90‐Arp2/3 nucleated filaments or 2 μl of spontaneously nucleated actin filaments were mixed with 10 nM Capping protein, 1 μΜ 15% Alexa‐568 actin with or without 0.5 μΜ GMF, and flowed into the chamber immediately, using an F‐buffer supplemented with 0.3% methylcellulose 4,000 cP. The fraction of actin filaments with an elongating pointed end is quantified at time *t* = 350 s after solution mixing. To study the uncapping effect of VCA, the same experiment was done except that we used 5 nM Arp2/3, 2 μM SPIN90 Cter, and 0.5 μM 15% Alexa‐488 actin to generate SPIN90‐Arp2/3‐nucleated actin filaments to minimize the amount of Arp2/3 in the system.

#### Arp2/3‐mediated branches assays

##### Dissociation of branches by VCA motifs, cortactin, and forces

The coverslip surface of a microfluidics chamber was functionalized by exposing it to 2.5 pM spectrin‐actin seeds for 5 min, followed by a passivation with a solution containing 3% BSA for 5 min. Mother filaments were grown from seeds by exposing them to 0.7 μM 15% Alexa‐488 actin. Mother filaments were then exposed to 0.15 μM unlabeled actin for 1 min. To generate the branches, the mother filaments are sequentially exposed to a solution of 30 nM Arp2/3 and 0.25 μM VCA for exactly 2 min, followed by a solution of 0.5 μM 15% Alexa‐568 actin for 5 min in order to obtain branches in a different color than the one of the mother filaments. Arp2/3‐generated branches were then exposed to 0.15 μM 15% Alexa‐488 G‐actin in order to maintain their length constant, and to various concentrations of regulatory proteins. Different concentrations of the different VCA motifs were used (Fig 2D–F), as well as 0.5 μΜ N‐WASP‐VCA with various concentration of actin (Fig EV3B), different concentrations of N‐WASP‐CA (Fig EV3D), and cortactin (Fig 7A and Appendix Fig S3). The survival fractions of branches over time were fitted with a single exponential function that yields the dissociation rates of branches for each condition. The dissociation rates were plotted versus the concentration of VCA, and a fit using the Michaelis–Menten equation was used to obtain the affinity constant and maximum dissociation rate (Fig 2F).

Various pulling forces can be applied to actin branches as described in Pandit *et al* (2020). The dissociation of actin branches is measured under a range of constant pulling forces (filaments are exposed to 0.15 μM G‐actin to maintain their length constant) in the presence or absence of 1 μM VCA or 1 μM GMF (Fig 5A–D). Likewise, the dissociation rate of aged actin branches is performed by exposing branches to 0.15 μM G‐actin for 20 min prior to monitoring their dissociation (Fig 6).

##### Branching efficiency of different NPFs (VCA motif of NWASP WASP and WASH)

The coverslip surface of a microfluidics chamber was functionalized by exposing it to 4 pM spectrin‐actin seeds for 5 min, followed by a passivation with a solution containing 5% BSA for 5 min. Mother filaments were polymerized by flowing 0.7 μM 10% Alexa‐488 actin. Then, for 90 s, we exposed the mother filaments to a solution of 20 nM Arp2/3, 50 nM VCA, and 0.4 μM 10% Alexa‐568 actin to generate branches. We next flowed in a solution of 0.5 μM 10% Alexa‐568 actin for 90 s to further grow the branches. After that time, branch densities were determined by counting the number of branches on mother filaments, and measuring the length of mother filaments.

#### GST pull‐down assays

Prior to each pull‐down assay, the Glutathione Sepharose 4B resin (GE Healthcare) was incubated with 5% BSA for 5 min and washed three times with 500 μl GST pull‐down buffer containing 50 mM Tris–HCl pH 7.0, 1 mM DTT, 50 mM KCl, and 5% glycerol. One‐hundred microliter of 2 μM Glutathione Sepharose 4B attached GST SPIN90 full length was mixed with 250 nM Arp2/3. A concentration gradient (0, 0.5, 2, and 4 μM) of GMF or GFP‐VCA was mixed with the loaded resin and allow to incubate for 1 h at 4°C. The resin was then washed three times with 300 μl of GST pull‐down buffer. Resin‐attached proteins were eluted with 50 μl of 20 mM GSH. The sample was separated by SDS–PAGE for western blot analysis. Anti‐ArpC2 (Sigma, HPA008352, Rabbit) was used to detect Arp2/3. GFP Monoclonal Antibody (Invitrogen, A‐11120, Mouse) was used to detect the GFP‐VCA.

The negative control experiment was done in the same way except 50 μl of 2 μM Glutathione Sepharose™ 4B attached GST was mixed with 250 nM Arp2/3, with or without 4 μM GMF or GFP‐VCA for 1 h at 4°C.

### Data analysis

Fiji software (Schindelin *et al*, 2012) was used to analyze images manually. No blinding procedure was applied. For nucleation assays, we counted all the filaments that appeared in a randomly chosen region (cropped field of view). For debranching and detachment experiments, filaments were chosen randomly without prior knowledge of their behavior. Filaments or branches that appeared to stick to the coverslip surface, or that were difficult to monitor because they overlapped with other filaments, were excluded from the analysis, regardless of how rapidly they detached or debranched. On rare occasions, the mother filaments severed and disappeared (flowed away) before debranching occurred, and such events were treated as censoring events in the Kaplan–Meier formalism.

#### Survival fraction analysis

We used the Kaplan–Meier method (“survival” package in R) to estimate and plot the survival fractions of the different populations probed in microfluidics assays. Data were fitted with a single exponential decay function to obtain the experimental detaching or debranching rates.

In nucleation experiments (Fig 1), the total number of nucleation sites in the field of view (plateau) was determined by the exponential fit of the curve, and used to normalize the number of nucleated filaments.

#### VCA and GMF protein affinity for SPIN90‐Arp2/3 or Arp2/3 branches

The binding affinity curves in Figs 2 and 3 were derived using the Michaelis–Menten equation: k_obs(x) = k_max.x/(K_D_ + x), where x is the protein concentration in solution, k_max is the maximum apparent rate of the reaction, and K_D_ the dissociation constant of the pseudo‐first‐order reaction.

#### Statistical significance

To compare the survival distributions of two samples, we computed the *P*‐value from the log‐rank test (the standard test for survival time analysis), using the “survival” package in R.

In Fig 4, to assess the significance of the statistical difference between fractions of SPIN90‐Arp2/3 complexes that re‐nucleated a filament in different conditions (Fig 4B and C), or the fraction of elongating pointed ends after their detachment from SPIN90‐Arp2/3 (Fig 4E and F), *P*‐values were calculated based on an unpaired *t*‐test (using R) because experiment repeats were independent.

## Author contributions

**Luyan Cao:** Conceptualization; formal analysis; investigation; writing – original draft; writing – review and editing. **Foad Ghasemi:** Formal analysis; investigation. **Michael Way:** Resources; funding acquisition; writing – review and editing. **Antoine Jégou:** Conceptualization; supervision; funding acquisition; writing – original draft; project administration; writing – review and editing. **Guillaume Romet‐Lemonne:** Conceptualization; supervision; funding acquisition; writing – original draft; project administration; writing – review and editing.

## Disclosure and competing interests statement

The authors declare that they have no conflict of interest.

## Supporting information



Appendix

Expanded View Figures PDF

PDF+

Source Data for Figure 1

Source Data for Figure 2

Source Data for Figure 3

Source Data for Figure 4

Source Data for Figure 5

Source Data for Figure 6

Source Data for Figure 7

## Data Availability

The data used to make the figures can be found in the Source Data files. This study includes no data deposited in external repositories.
