# Supplementary material for: Regulation of branched versus linear Arp2/3‐generated actin filaments
Source: EMBO J. 2023 Mar 20;42(9):e113008. doi: 10.15252/embj.2022113008 (PMC10152144; doi:10.15252/embj.2022113008)
Supplement: Supplementary file 1 — Appendix [file EMBJ-42-e113008-s008.pdf]

APPENDIX

Regulation of branched versus linear Arp2/3-generated actin filaments
Cao et al.

Contains Appendix Figures S1-S4

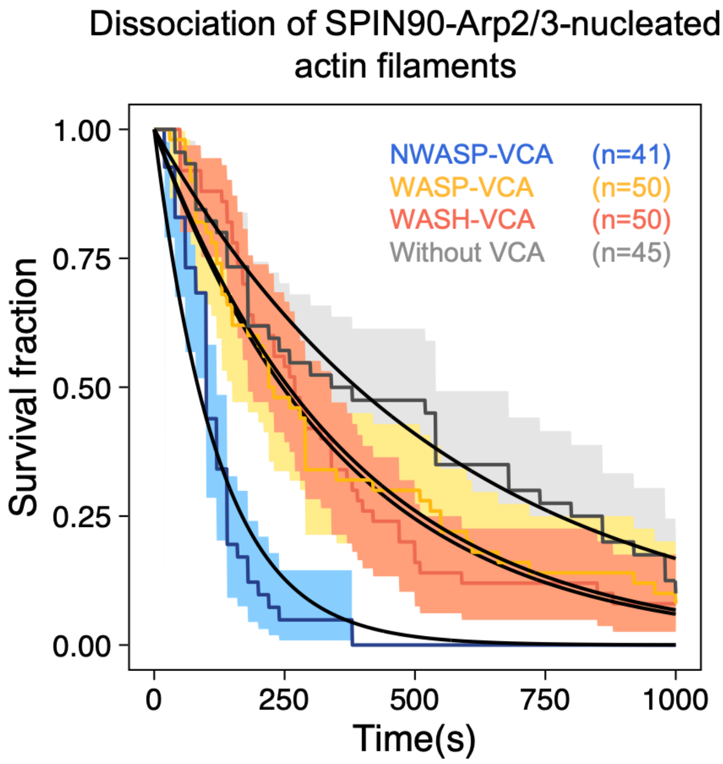

Appendix Figure S1. Detachment of SPIN90-Arp2/3-nucleated filaments exposed to VCA, during the nucleation experiment shown in figure 1.

Normalized number of filaments dissociated from the surface exposed to 2 µM G-actin (15% labeled with Alexa488) and 1 µM profilin, with 0 or 0.5µM of GST-VCA from different NPFs. Solid lines are exponential fits, yielding dissociation rates k_{off}=(1.8±0.6)x10^{-3} s^{-1} without VCA, and k_{off}=(7.9±1.9)x10^{-3}, (2.7±0.9)x10^{-3}, and (2.8±0.7)x10^{-3} s^{-1} with VCA from N-WASP, WASP and WASH, respectively. Indicated values of n are the number of filaments observed in each experiment. These experiments were repeated three times, with similar results.

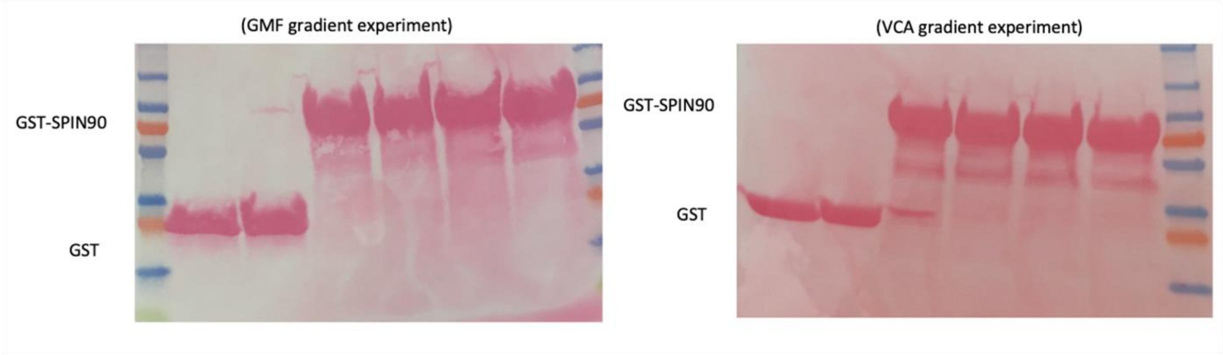

Appendix Figure S2. Amount of GST and GST-SPIN90 loaded on beads in the pull-down assays (Fig. 4G). In the experiments shown in Fig 4G, before passivating and adding with antibodies, the membrane was stained with Ponceau red.

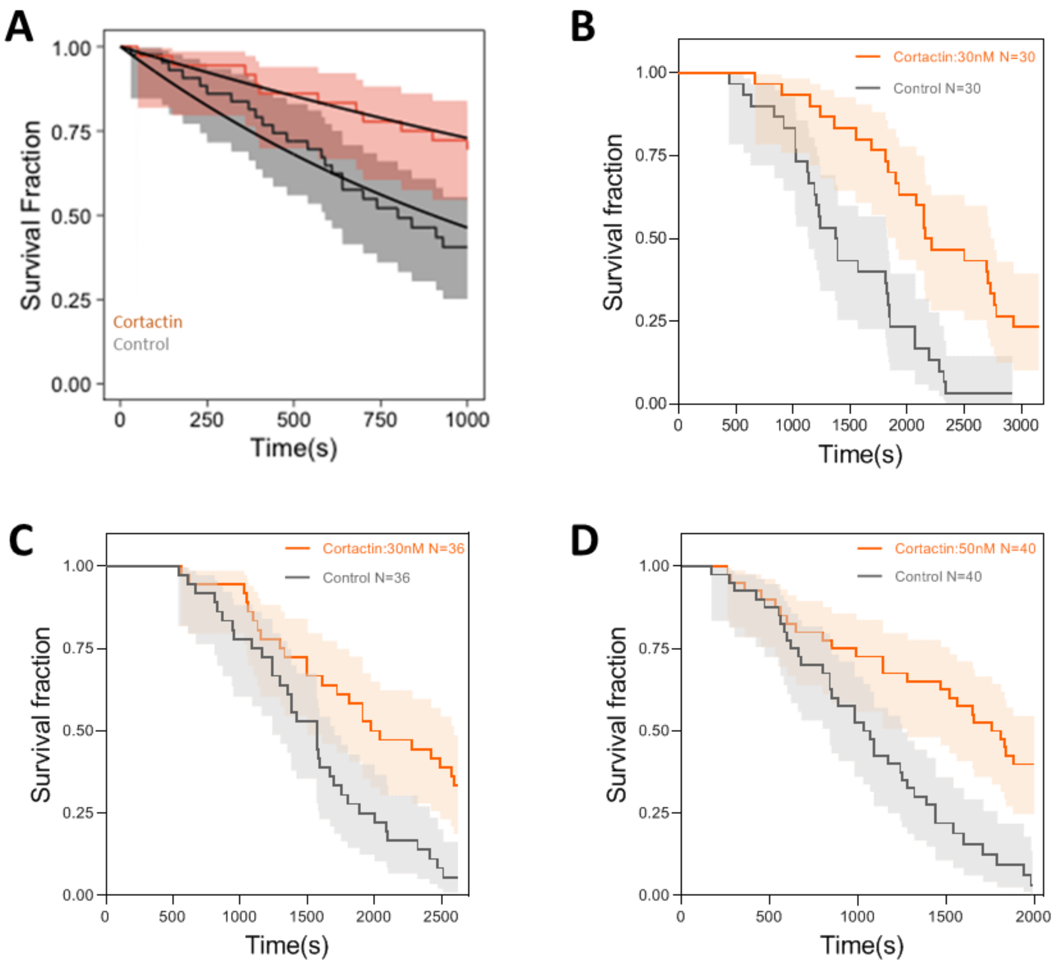

Appendix Figure S3. Cortactin slows down debranching.

A. The fraction of remaining branches, versus time, exposed to 0.15 µM G-actin (black) supplemented with 100 nM cortactin (red). The branch junctions were exposed to an average force of 0.2 pN. Black lines are exponential fits, yielding dissociation rates at $k_{off}=(7.7\pm1.4)\times10^{-4}\ s^{-1}$ without cortactin, and $k_{off}=(3.2\pm1.5)\times10^{-4}\ s^{-1}$ with 100 nM cortactin.

B. The fraction of remaining branches, versus time, exposed to 0.3 µM G-actin (black) supplemented with 30 nM cortactin (orange). In this case, actin keeps polymerizing during the measurement. Thereby, the forces applied on actin branches increase over time.

C. The fraction of remaining branches, versus time, exposed to 0.18 µM G-actin (black) supplemented with 30 nM cortactin (orange). The branch junctions were exposed to an average force of 0.38 pN, after being aged for 5 minutes in the absence of force.

D. The fraction of remaining branches, versus time, exposed to 0.15 µM G-actin (black) supplemented with 50 nM cortactin (orange). The branch junctions were exposed to an average force of 0.68 pN, after being aged for 10 minutes in the absence of force.

(A-D) The shaded areas represent 95% confidence intervals.

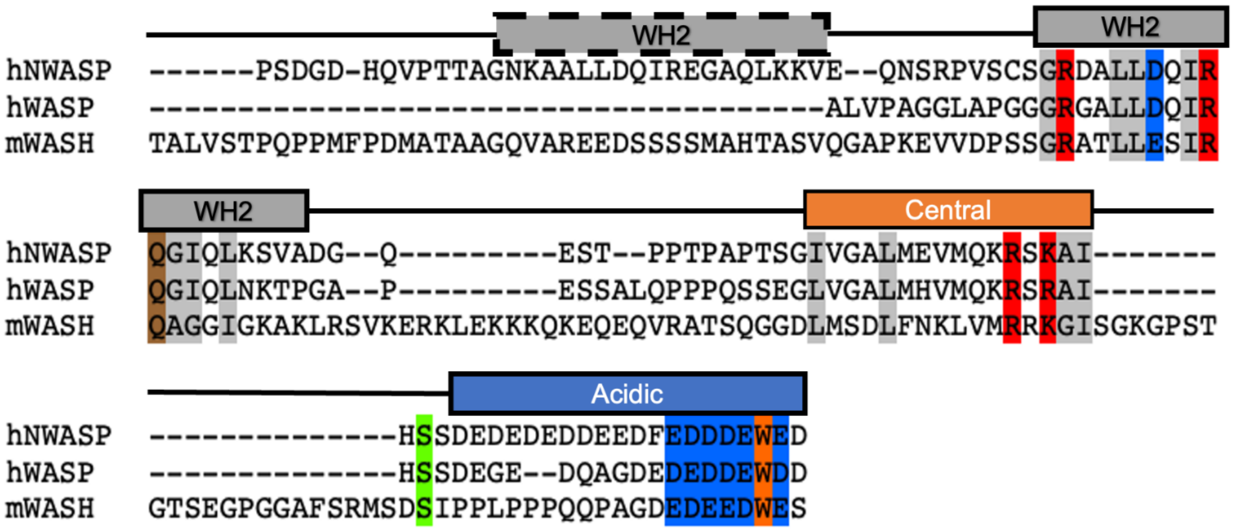

Appendix Figure S4. Sequence alignment of the VCA motifs used in this study. Domain diagram is shown at the top of the sequence (ref 1). The sequences of each conserved domains are aligned in Color Align Properties of the Sequence Manipulation Suite (ref 2). The identical amino acids among the three constructs are highlighted and colored according to the biochemical properties of the residue.

References:

1. Veltman D and Insall R, Molecular Biology of the Cell, 2010
2. Stothard P (2000) The Sequence Manipulation Suite: JavaScript programs for analyzing and formatting protein and DNA sequences. Biotechniques 28:1102-1104.
