# Supplementary material for: Regulation of branched versus linear Arp2/3‐generated actin filaments
Source: EMBO J. 2023 Mar 20;42(9):e113008. doi: 10.15252/embj.2022113008 (PMC10152144; doi:10.15252/embj.2022113008)
Supplement: Supplementary file 2 — Expanded View Figures PDF [file EMBJ-42-e113008-s005.pdf]

Expanded View Figures

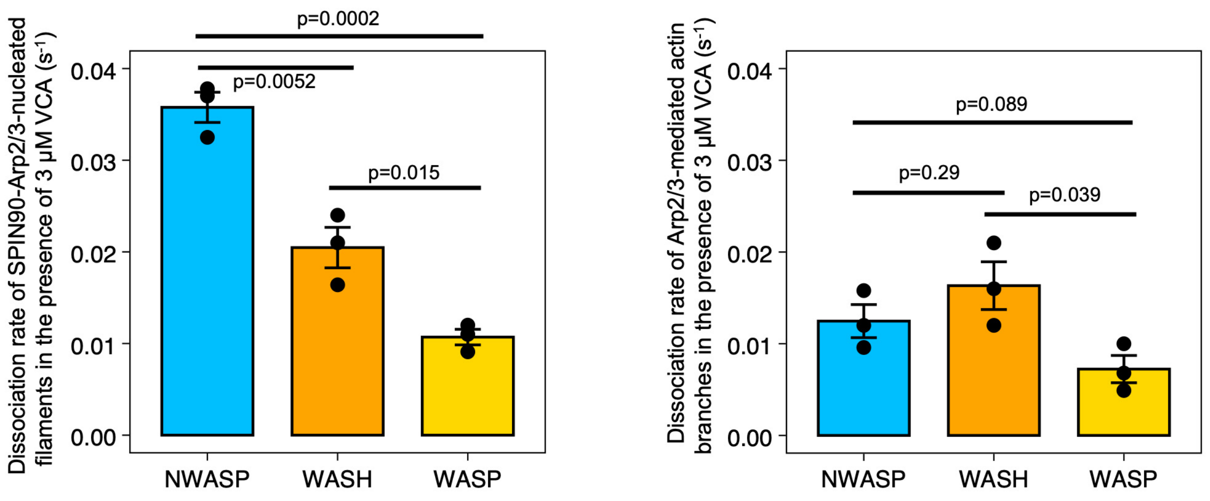

Figure EV1. Comparing the VCA motifs from different NPFs in their ability to destabilize linear filaments nucleated by SPIN90-Arp2/3 (left) or Arp2/3-mediated branches (right).

The dissociation rates of Arp2/3-nucleated filaments exposed to nearly saturating amounts (3 μM) of VCA from different NPFs were measured, as in Fig 2. The histogram represents the average koff of three independent measurements (black dots) and the error bars represent the standard deviation. The dissociation rate of SPIN90-Arp2/3-nucleated filaments is 0.037 ± 0.003, 0.022 ± 0.002, and 0.011 ± 0.001 s^{-1}, for N-WASP, WASH, and WASP, respectively. The dissociation rate of Arp2/3-mediated actin branches is 0.012 ± 0.003, 0.016 ± 0.005, and 0.007 ± 0.003 s^{-1}, for N-WASP, WASH, and WASP, respectively. The *P*-values were determined with an unpaired *t*-test.

© 2023 The Authors

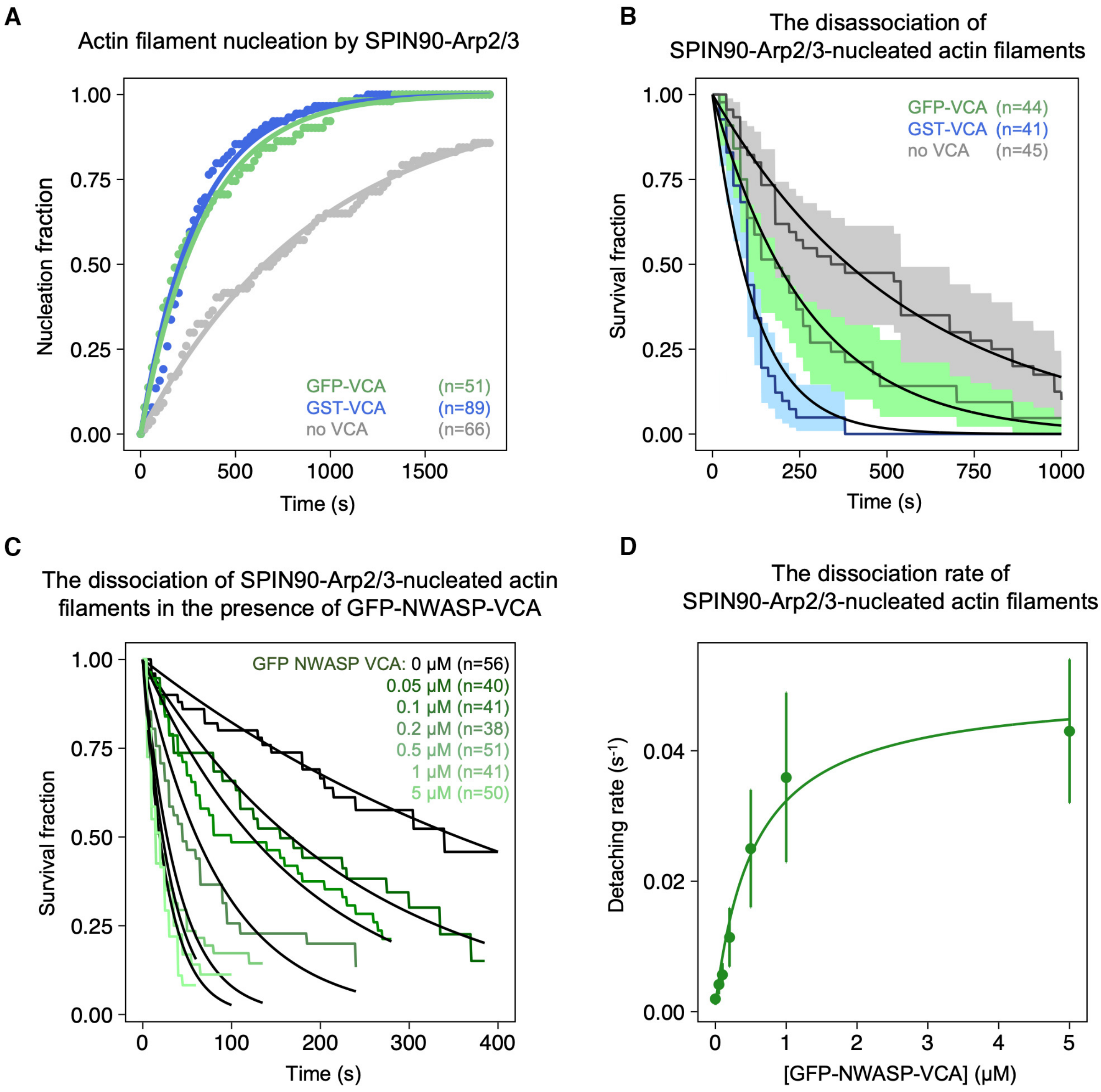

Figure EV2. Impact of VCA dimerization.

A Normalized number of filaments nucleated over time, from SPIN90-Arp2/3 exposed to 2 μM G-actin (15% labeled with Alexa488) and 1 μM profilin, with 0 or 0.5 μM of GST-N-WASP-VCA or GFP-N-WASP-VCA. Solid lines are exponential fits, yielding nucleation rates knuc = (1.06 ± 0.03) × 10^{-3} s^{-1} without VCA, and knuc = (3.23 ± 0.08) × 10^{-3} s^{-1} and (3.02 ± 0.04) × 10^{-3} s^{-1} with GST-N-WASP-VCA and GFP-N-WASP-VCA, respectively. Indicated values of *n* are the number of filaments observed in each experiment. These experiments were repeated three times, with similar results.

B Detachment of SPIN90-Arp2/3-nucleated filaments during the nucleation experiment shown in panel A. Solid lines are exponential fits, yielding dissociation rates koff = (1.8 ± 0.6) × 10^{-3} s^{-1} without VCA, and koff = (7.9 ± 1.9) × 10^{-3} s^{-1} and (3.7 ± 1.4) × 10^{-3} s^{-1} with GST-N-WASP-VCA and with GFP-N-WASP-VCA, respectively. Indicated values of N are the number of filaments observed in each experiment.

C The fraction of filaments still attached to the surface, versus time, for different concentrations of GFP-NWASP-VCA. Black lines are exponential fits. Indicated values of n are the number of filaments observed in each experiment. Each experiment was repeated twice (technical replicates), yielding similar results.

D Detachment rates determined by exponential fits of survival curves (in C) as a function of the concentration of VCA motifs from different NPFs. The error bars result from fits of the 95% confidence interval in the survival curves. The data are fitted by a Michaelis–Menten equation, resulting in KD = 0.56 ± 0.11 μM and Vmax = 0.049 ± 0.0034 s^{-1}.

© 2023 The Authors

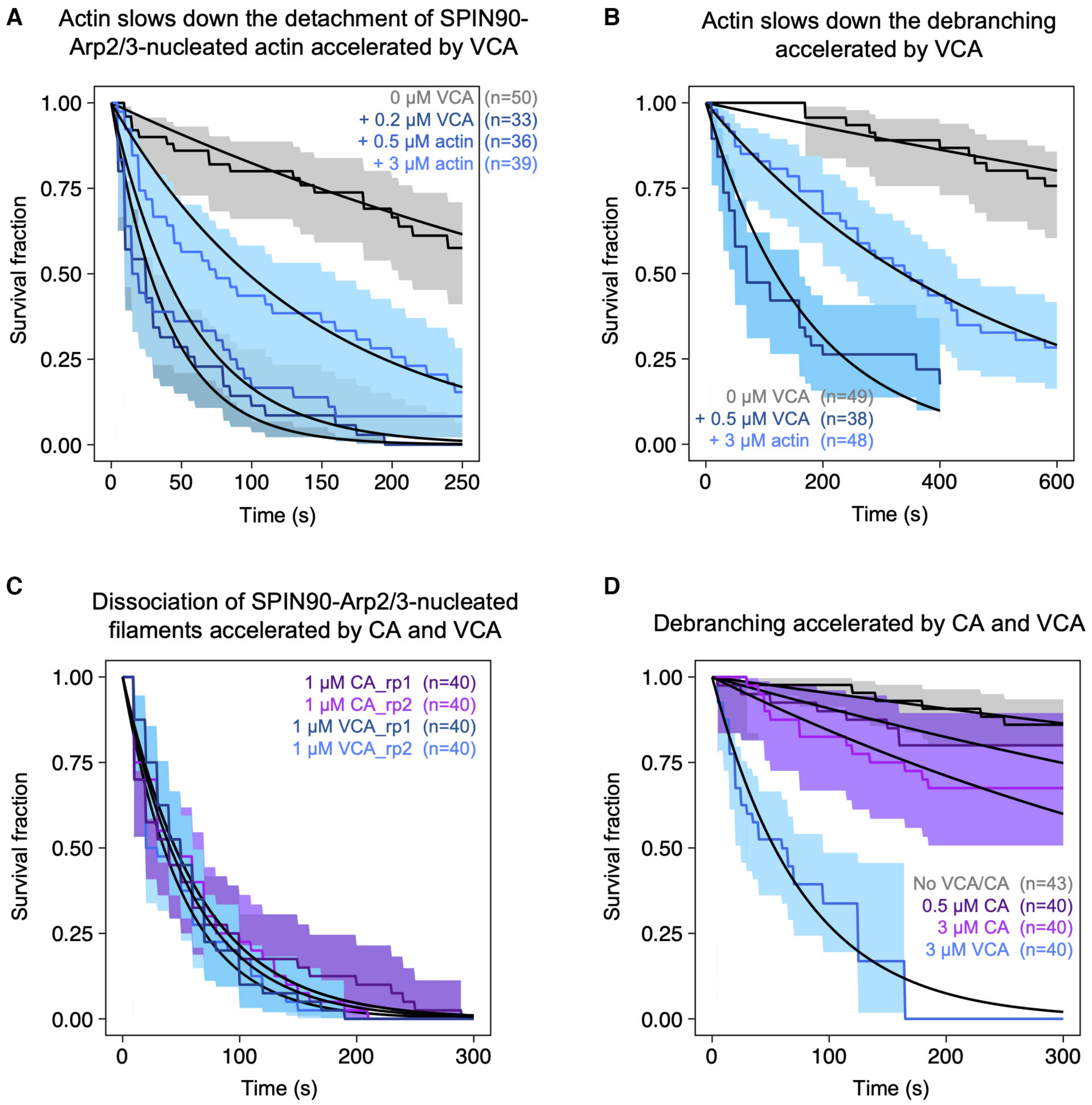

Figure EV3. The presence of G-actin inhibits the destabilizing action of VCA, but the V domain is not required to enhance the detachment of SPIN90-Arp2/3-nucleated filaments.

A The fraction of filaments still attached to the surface versus time, exposed to buffer, or to 0.2 µM VCA with different concentrations of G-actin. Black lines are exponential fits, yielding dissociation rates koff = (2.1 ± 0.8) × 10^{-3} S^{-1} without VCA, and koff = (2.6 ± 0.7) × 10^{-2} s^{-1} with 0.2 µM VCA, (1.8 ± 0.8) × 10^{-2} s^{-1} with 0.2 µM VCA plus 0.5 µM G-actin, and (0.76 ± 0.28) × 10^{-2} s^{-1} with 0.2 µM VCA plus 3 µM G-actin.

B The fraction of actin branches still attached to the mother filaments versus time, exposed to buffer, or to 0.5 µM VCA with 0.15 µM G-actin, or to 0.5 µM VCA with 3 µM G-actin. Black lines are exponential fits, yielding dissociation rates koff = (0.37 ± 0.7) × 10^{-3} s^{-1} without VCA, and koff = (5.8 ± 2.3) × 10^{-3} s^{-1} with 0.5 µM VCA, and (2.1 ± 0.7) × 10^{-3} s^{-1} with 0.5 µM VCA plus 3 µM G-actin. The shaded areas represent 95% confidence intervals.

C The fraction of filaments still attached to the surface versus time, exposed to 0.2 µM G-actin supplemented with 1 µM VCA or 1 µM CA. Black lines are exponential fits, yielding dissociation rates koff = (1.7 ± 0.02) × 10^{-2} s^{-1} and koff = (2.0 ± 0.02) × 10^{-2} s^{-1} for the two repeats with VCA; koff = (1.5 ± 0.02) × 10^{-2} s^{-1} and koff = (1.5 ± 0.02) × 10^{-2} s^{-1} for the two repeats with CA.

D The fraction of actin branches still attached to the mother filaments versus time, exposed to buffer, or to 0.15 µM actin supplemented with 0.5 µM CA, 3 µM CA, or 3 µM. Black lines are exponential fits, yielding dissociation rates koff = (5.0 ± 0.04) × 10^{-4} s^{-1} without buffer, and koff = (1.0 ± 0.01) × 10^{-3} s^{-1} with 0.5 µM CA, (1.8 ± 0.01) × 10^{-3} s^{-1} with 3 µM CA, and (1.3 ± 0.02) × 10^{-2} s^{-1} with 3 µM VCA.

Data information: In the text above, uncertainty intervals correspond to the standard errors. In the figures, the shaded areas represent 95% confidence intervals. Indicated values of n are the number of filaments monitored in each experiment. The flowing solutions applied a weak force to the filaments (< 1 pN) and to the branches (0.2 pN).

© 2023 The Authors

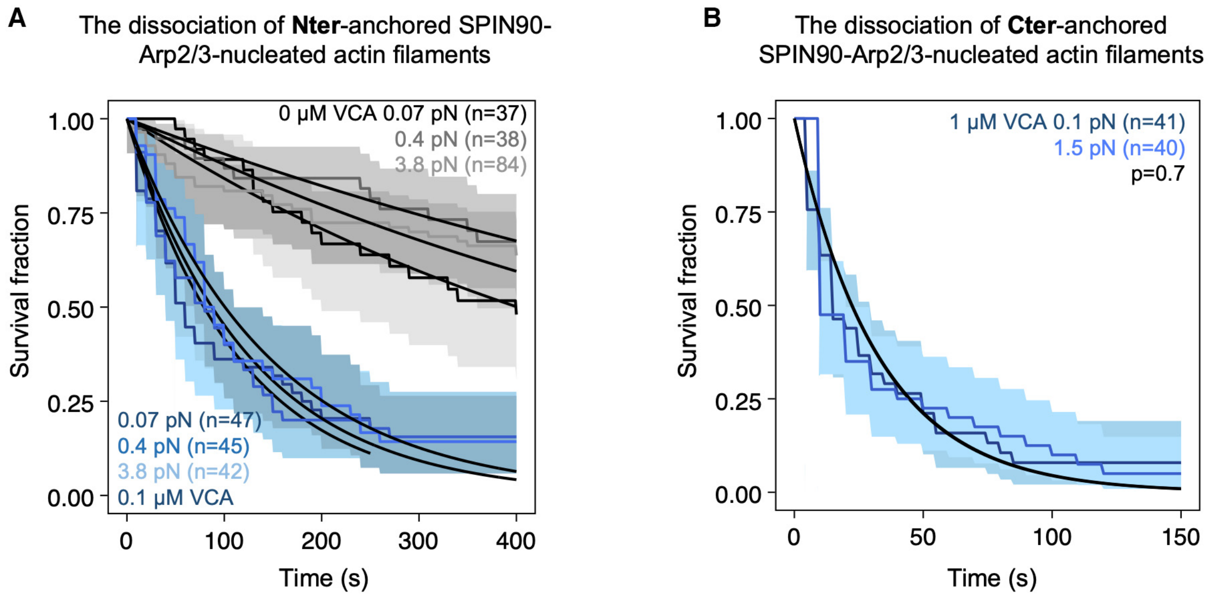

Figure EV4. Anchoring SPIN90 by its C- or N-terminus has no impact on its behavior.

A Survival fractions for SPIN90-Arp2/3-nucleated filaments exposed to different forces in the microfluidics chamber, while being exposed to 0.15 μM G-actin alone or with 0.1 μM GFP-VCA. In this case, SPIN90 was anchored on the surface through its Nter-GST-tag. Black lines are exponential fits, yielding dissociation rates at 0.07 pN force, koff = (1.6 ± 0.6) × 10^{-3} s^{-1} without VCA, and koff = (7.5 ± 3.3) × 10^{-3} s^{-1} with 0.1 μM VCA; at 0.4 pN force, koff = (1.2 ± 0.5) × 10^{-3} s^{-1} without VCA, and koff = (7.4 ± 3.5) × 10^{-3} s^{-1} with 0.1 μM VCA; 3.8 pN, koff = (1.3 ± 0.4) × 10^{-3} s^{-1} without VCA, and koff = (6.9 ± 2.4) × 10^{-3} s^{-1} with 0.1 μM VCA. The shaded areas represent 95% confidence intervals.

B Survival fractions for SPIN90-Arp2/3-nucleated filaments exposed to 1 μM VCA. In this case, SPIN90 was anchored on the surface through its Cter-His-tag. Black lines are exponential fits, yielding dissociation rates koff = (3.1 ± 1.5) × 10^{-2} s^{-1} at 0.1 pN force and koff = (3.1 ± 1.3) × 10^{-2} s^{-1} at 1.5 pN force. The shaded areas represent 95% confidence intervals.

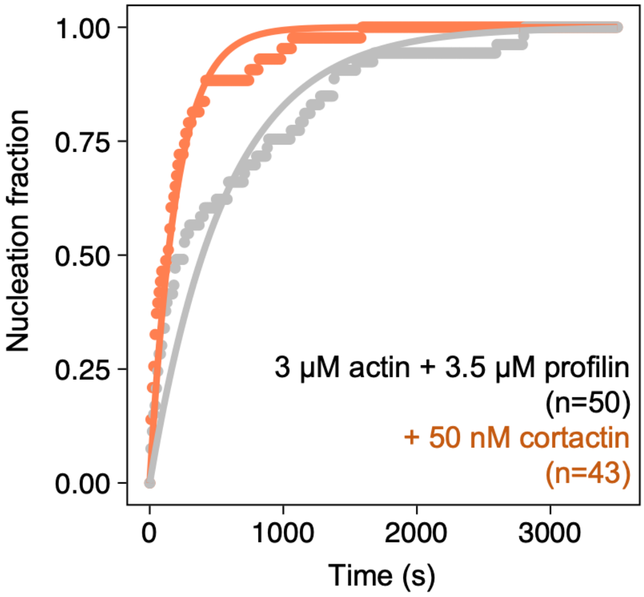

Figure EV5. SPIN90-Arp2/3 filament nucleation is accelerated by cortactin.

Normalized number of filaments nucleated over time, from SPIN90-Arp2/3 exposed to 3 μM G-actin (15% labeled with Alexa488) and 3.5 μM profilin, with 0 or 50 nM of cortactin. Solid lines are exponential fits, yielding nucleation rates knuc = (2.0 ± 0.04) × 10^{-3} s^{-1} without cortactin, and knuc = (5.3 ± 0.08) × 10^{-3} s^{-1} with cortactin. Indicated values of n are the number of filaments observed in each experiment.

 © 2023 The Authors
