# Supplementary figures and images for: Regulation of branched versus linear Arp2/3‐generated actin filaments
Source: EMBO J. 2023 Mar 20;42(9):e113008. doi: 10.15252/embj.2022113008 (PMC10152144; doi:10.15252/embj.2022113008)

Source Data for Figure 4 G-H

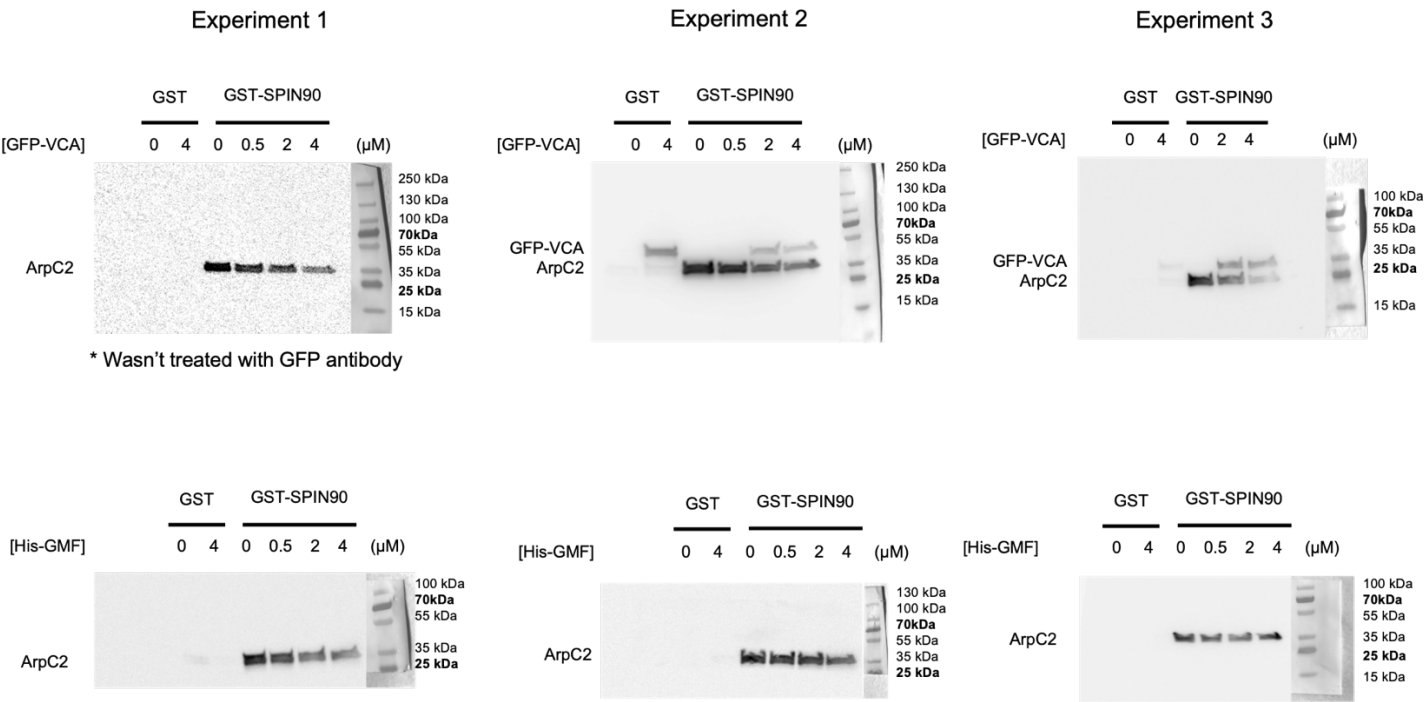

Supplement: Supplementary file 7 — Source Data for Figure 4 [file EMBJ-42-e113008-s006.zip › EMBOJ-2022-113008_SourceDataForFigure4GHI.pdf]
