# Supplementary material for: Regulation of branched versus linear Arp2/3‐generated actin filaments
Source: EMBO J. 2023 Mar 20;42(9):e113008. doi: 10.15252/embj.2022113008 (PMC10152144; doi:10.15252/embj.2022113008)
Supplement: Supplementary file 11 [file EMBJ-42-e113008-s010.pdf]

THE EMBO JOURNAL

Regulation of branched versus linear Arp2/3-generated actin filaments

Luyan Cao, Foad Ghasemi, Michael Way, Antoine JEGOU, and Guillaume Romet-Lemonne
DOI: 10.15252/embj.2022113008

Corresponding author(s): Guillaume Romet-Lemonne (romet@ijm.fr) , Antoine JEGOU (antoine.jegou@ijm.fr), Luyan Cao (luyan.cao@crick.ac.uk)

Review Timeline:

Transfer from Review Commons:	7th Nov 22
Editorial Decision:	24th Nov 22
Revision Received:	1st Feb 23
Accepted:	1st Mar 23

Review COMMONS

Editor: Ieva Gailite

Transaction Report: This manuscript was transferred to The EMBO JOURNAL following peer review at Review Commons.

Review #1

1. Evidence, reproducibility and clarity:

Evidence, reproducibility and clarity (Required)

An exciting development in our knowledge about how the Arp2/3 complex controls the assembly of actin networks has come from the discovery that in addition to forming branched networks, Arp2/3 can nucleate linear filaments when it is activated by WISH/DIP/SPIN90. However, despite some excellent work largely done by the Nolen lab in yeast, many questions remain about how Arp2/3-mediated assembly of branched vs. linear actin filament. This is especially true in the complex environment of cells, were synergy and competition of different actin networks is used to control biological processes. Knowing the biochemical and physical properties of these different Arp2/3 assemblies will be key to figuring out how they work in cells. Here Cao et al. use an elegant microfluidics based single filament assay system to perform a comparative analysis of the stability of linear and branched Arp2/3 networks. They find interesting differences in how they respond to stabilizing and destabilizing factors. The most striking differences happens when force or aging is applied- both cause debranching of branched networks but have little effect on Spin90-Arp2/3 nucleated filaments.

Major comments:

As a comparative study on the stability of branched vs. linear Arp2/3 nucleated filaments, this manuscript is fairly complete. The key conclusions are well supported by rigorous experiments which can be reproduced by others based on the information provided. However, I am not seeing explicit information on performing biological replicates. This should be included in the manuscript. The use of statistics is largely fine; however I question the use of one statistical test on one figure (see minor comments below).

I would not ask for additional experiments at this time. However, there is an analysis that would be important for interpreting the authors' claims- branch/filament length at the time of dissociation or destabilization of Arp2/3. This would help address if there was a physical tipping point for each type of structure that could explain potential differences they see. The authors should already have this data and the time to complete it would be negligible in delaying publication.

One additional major comment is that the manuscript's title and abstract hint that this paper explores the differences in nucleation of branched vs. linear filaments by Arp2/3. However, the only figure that deals explicitly with nucleation in the paper is Figure 1, which is really just a confirmation that the mammalian proteins used in this study perform similarly to their yeast homologues (Balzer et al, Current Biology 2019). The authors might think about rewording the title/abstract to better reflect that paper really explores the differences in the stability of the two networks

Minor comments:

1 in 12 men and 1 in 200 women are red/green colorblind. Please change the coloring of the schematics and images so that they can be easily seen by all people. This is especially true of the schematics, which are important for understanding exactly what each assay is measuring.

The Introduction is a bit choppy and unfocused. It was difficult to deduce exactly where the paper was going from it. Please consider re-writing it for better clarity. The Discussion on the other hand was fantastic. Great job on interpreting your results in a larger context.

Many figures- while the use of different lightness values of the same color is appreciated in conveying different concentrations of reagents used, there were several instances where it was very hard to read the one on the very bottom (ex. 2B, E; 3A; 5C, G).

Figure 1- since this is a confirmation of previous results performed using the same proteins from other species, the title should reflect that (ex. VCA domains accelerate the nucleation of filaments by mammalian SPIN90-Arp2/3). Also, to me this figure is supplementary to the main message of the paper. The authors might think of moving it to Supplementary Information.

Figure 1- If the goal was to verify that G-actin recruitment by VCA was important for Spin90-Arp 2/3 nucleation by performing a competition experiment with profilin, why was the concentration of G-actin AND profilin increased between the experiments in 1B vs. 1C. It makes it hard to directly compare the results.

Figure 4B-F- Here, it would be nice to see the distribution of all the individual results, which are hidden by the bar graph. Additionally, the Chi-square test is not the appropriate test for evaluating statistical significance between multiple groups. ANOVA followed by an appropriate post hoc test should be used here.

Figure 4G- Please quantify and show reproducibility.

Figure 5- the piconewton forces used for these experiments is in line with measured forces that are applied to actin in cells (ex. Mehida et al, Nature Cell Biology 2021; Jiang et al, Nature 2003). The text would benefit if this was explicitly stated.

2. Significance:

Significance (Required)

The real significance of this work is in characterizing the differential stabilities of linear vs. branched Arp2/3 filaments in response to actin-binding proteins, mechanical stress, and aging. While both types of filaments respond similarly to actin-binding proteins, with nuanced differences, the most striking results came from applied force and aging experiments, with Spin90-Arp2/3 filaments being much more resistant to both. This has some very interesting implications for how these two types of assemblies might synergize in cells. Additionally, the results also have some exciting implications for the pointed-end regulation of actin filaments, which is still poorly understood in complex systems. Since the manuscript is A) more of a survey study on the factors that influence filament stability that does not go particularly deep into any particular mechanism of regulation and B) has no direct applicability to how the physical properties of branched and linear Arp2/3 nucleated actin filaments influence actin network activity in cells, the audience will likely by limited to actin enthusiasts. However, the work is still important in both what it reveals and implies.

3. How much time do you estimate the authors will need to complete the suggested revisions:

Estimated time to Complete Revisions (Required)

(Decision Recommendation)

Less than 1 month

4. *Review Commons* values the work of reviewers and encourages them to get credit for their work. Select 'Yes' below to register your reviewing activity at [Web of Science Reviewer Recognition Service]() (formerly Publons); note that

the content of your review will not be visible on Web of Science.

Web of Science Reviewer Recognition

Yes

Review #2

1. Evidence, reproducibility and clarity:

Evidence, reproducibility and clarity (Required)

The quantitative analysis can be improved. It appears that most of the data results from single experiments, with rate values and errors resulting from fitting of single experiments without repetitions. In Fig. 1C legend (p.5) the authors state "These experiments were repeated three times, with similar results", but the data is not used in the analysis and other experiments do not mention this point. This is particularly important for comparisons among different VCAs that are rather similar in nature. In Fig. 1B. N-WASP is more efficient in nucleating SPIN90-Arp2/3 complex-linear filaments followed by WASP and then WASH. In Fig. 2 B,C, N-WASP is the most effective in dissociating SPIN90-Arp2/3 complex linear filaments followed by WASH and then WASP. But in Fig. 2 E,F, WASH is by far the most effective in dissociating branches followed by N-WASP and then WASP. Therefore, the conclusion in the Discussion (p.12) "While these regulatory proteins similarly affect branched and linear Arp2/3-generated filaments, they do so with clear quantitative differences" is not supported by quantification. To remedy this problem the authors should include at least 3 repeats of each experiment in data analysis. Also, they could include an analysis of sequence differences among VCAs and discuss how these may correlate with the observed differences. For instance, one WH2 in WASP vs. two in N-WASP.

2. Significance:

Significance (Required)

Arp2/3 complex is a 7-protein complex implicated in actin filament nucleation and branching. Arp2/3 complex-nucleated branched networks are found at several

locations in cells and are responsible for processes such as cell motility.

Cao et al. compare the effect of several proteins on the filament nucleation activity of Arp2/3 complex, and the stabilization or destabilization of actin filament branches as well as linear actin filaments nucleated by SPIN90-Arp2/3 complex. The proteins tested include the VCA regions of three NPFs (N-WASP, WASP, and WASH) that activate Arp2/3 complex, GMF (a debranching protein) and cortactin (a branch stabilizing protein). For the most part, the study uses a single method, microfluidics-TIRF microscopy.

The main findings are:

1. VCA domains enhance nucleation of linear filaments by SPIN90-Arp2/3 complex in the presence of actin monomers.
2. However, VCA domains can also destabilize existing SPIN90-Arp2/3 complex linear filaments and branches, and this effect depends on the presence of of V-domain (WH2 domain that binds actin monomers).
3. The debranching factor GMF also destabilizes SPIN90-Arp2/3 complex linear filaments. Both GMF and VCA generate free pointed ends by dissociating Arp2/3 complex from pointed ends and SPIN90.
4. SPIN90-Arp2/3 complex linear filaments are less susceptible to force and aging than filament branches.
5. Cortactin stabilizes SPIN90-Arp2/3 complex linear filaments to higher degree than it does branches.

These are novel and very interesting new observations of significant interest to the actin cytoskeleton field. Therefore, I recommend publication of this paper in EMBO J. I have one recommendation and one suggestion for improvement:

Major:

1. The quantitative analysis can be improved. It appears that most of the data results from single experiments, with rate values and errors resulting from fitting of single experiments without repetitions. In Fig. 1C legend (p.5) the authors state "These experiments were repeated three times, with similar results", but the data is not used in the analysis and other experiments do not mention this point. This is particularly important for comparisons among different VCAs that are rather similar in nature. In Fig. 1B. N-WASP is more efficient in nucleating SPIN90-Arp2/3 complex-linear filaments followed by WASP and then WASH. In Fig. 2 B,C, N-WASP is the most effective in dissociating SPIN90-Arp2/3 complex linear filaments followed by WASH and then WASP. But in Fig. 2 E,F, WASH is by far the most effective in dissociating

branches followed by N-WASP and then WASP. Therefore, the conclusion in the Discussion (p.12) "While these regulatory proteins similarly affect branched and linear Arp2/3-generated filaments, they do so with clear quantitative differences" is not supported by quantification. To remedy this problem the authors should include at least 3 repeats of each experiment in data analysis. Also, they could include an analysis of sequence differences among VCAs and discuss how these may correlate with the observed differences. For instance, one WH2 in WASP vs. two in N-WASP.

Minor:

2. In GST-pull-down experiments (Fig. 4G), the amount of Arp2/3 complex bound is analyzed by Western, which is rather unprecise. Is the amount of Arp2/3 complex so little that it cannot be quantified using regular SDS-PAGE? If that is the case, this would suggest rather low affinity of SPIN90 for Arp2/3 complex. How does this affect the proposed mechanism and experiments in the microfluidics chamber?

3. How much time do you estimate the authors will need to complete the suggested revisions:

Estimated time to Complete Revisions (Required)

(Decision Recommendation)

Less than 1 month

4. *Review Commons* values the work of reviewers and encourages them to get credit for their work. Select 'Yes' below to register your reviewing activity at Web of Science Reviewer Recognition Service (formerly Publons); note that the content of your review will not be visible on Web of Science.

Web of Science Reviewer Recognition

No

Review #3

1. Evidence, reproducibility and clarity:

Evidence, reproducibility and clarity (Required)

Summary:

In this study, Cao and collaborators investigate the biochemical and mechanical differences between branched actin filaments nucleated by WASP-activated Arp2/3 complex and linear actin filaments nucleated by SPIN90-activated Arp2/3 complex. They use TIRF microscopy in a microfluidic chamber to show that the mammalian proteins, SPIN90 and WASP (or N-WASP or WAVE), like their yeast homologues, co-activate Arp2/3 complex to nucleate linear actin filaments. Using the same assays, they find the surprising result that the VCA segment of WASP proteins destabilizes the interaction between SPIN90 and Arp2/3 complex in linear actin filaments nucleated by Arp2/3 complex. They then show that VCA also destabilizes actin filament branches. The remainder of the study explores the influence of branch stabilizing/destabilizing proteins or mechanical stress on the stability of the interaction between SPIN90 and Arp2/3 complex on the pointed end of the actin filament. They find that like branch junctions, SPIN90-bound Arp2/3 is destabilized at the end of linear filaments by GMF and stabilized by cortactin. However, unlike branch junctions, SPIN90-Arp2/3 complex is not destabilized on filament ends by piconewton forces or by aging. They conclude that SPIN90- versus VCA-activated Arp2/3 complex adopt similar but non-identical conformations.

Overall, the paper is well written and the experiments, which are very challenging, are rigorously executed. The biochemical results are convincing, novel and unexpected. However, the work could be strengthened by more strongly connecting the biochemical observations to biological implications. In addition, there are some interpretations/conclusions that seem somewhat weakly supported, and the authors should consider revising. Nonetheless, given the quality of the work and the importance of the system, this manuscript will appeal to a broad audience.

Comments on evidence, reproducibility, clarity and significance:

The differences in the stability of SPIN90-Arp2/3 on linear filaments verses branch junctions led the authors to conclude that SPIN90- versus VCA-activated complexes adopt similar yet non-identical conformations. There are two problems with this conclusion:

1. This conclusion rests on the idea that the biochemical differences can only be due to differences in the "ground state" active conformations of the complex. Another possible scenario would be that the active conformations are the same, but the transition state or intermediate state structures within the debranching reactions are different, thus changing the kinetics of the debranching reactions.
2. There are already structural data showing conformational differences between the Dip1-bound Arp2/3 complex on the end of a linear filament and Arp2/3 complex at a branch junction. While there are some caveats to comparisons of the structures (e.g., the Dip1 structure includes the fission yeast SPIN90 protein (Dip1) and the fission yeast Arp2/3 complex while the branch junction contains mammalian proteins), these data offer much stronger evidence that the active states adopt (somewhat) different conformations than the data presented here.

The authors make comparisons between the Fäβler branch junction structure and the Shaaban Dip1-Arp2/3-filament structure. The Fäβler branch junction structure is a low resolution structure (9 angstroms) and should be interpreted with caution (see below). A much higher resolution of a branch junction structure was recently solved (Ding et al, PNAS 2022) and should be used for comparisons between the structures.

Pg 14 - The authors say differences between ARPC3-Arp2 and ARPC5-Arp2 contacts in the two structures are likely to cause the differences in interactions with GMF and VCA. Two concerns with this statement are: 1.) The basis for the conclusion that the ARPC5-Arp2 contacts are different (in Fäβler, et al.) is not solid (see Ding, et al) and 2.) The analysis is vague. To reasonably conclude that differences in the contacts would influence GMF and VCA interactions would require mapping out the structural connection between the ARPC3-Arp2 interaction site and the GMF or VCA binding sites. If there is no obvious connection between these sites, the conclusion that the differences in the ARPC3-Arp2 interface cause differences in VCA and GMF binding should be far more circumspect.

Pg 6. "These observations suggest that the ability of VCA to destabilize Arp2/3-nucleated filaments relies on the availability of its V-domain." It's possible that G-actin binding to V blocks the CA from accessing the branch junction. Therefore, it seems important to test whether N-WASP-CA can destabilize Arp2/3-nucleated actin filaments.

Pg 1 - The authors state that "It thus appears that linear and branched Arp2/3-generated filaments respond similarly to regulatory proteins, albeit with quantitative differences". It is worth considering if one should make a blanket statement that linear and branched filaments respond similarly to regulatory proteins when they have tested

3 in total.

Pg 3 - "More generally, the stability of SPIN90-Arp2/3 at the pointed end, which is important to understand the reorganization and disassembly of actin filament networks, remains to be established." In some ways this statement not quite accurate because Balzer et al previously showed that Dip1-Arp2/3 complex is very stable at the pointed end. Is the question here whether that stability is also conserved in mammalian systems? If so, that should be more directly stated.

The observation that VCA accelerates debranching and SPIN90-Arp2/3 dissociation is very interesting. However, it is uncertain if this biochemical activity has biological relevance, given that once nucleation occurs, Arp2/3 complex will move away from the membrane. While the authors mention in the discussion that debranching by VCA could be relevant when the network is compressed near the membrane, this argument is not particularly strong. Are there ways to strengthen this argument, or find another impact this finding might have on our understanding of Arp2/3 complex regulation?

The observation that SPIN90+Arp2/3-nucleated filaments are not sensitive to piconewton forces is also very interesting. The authors focus on the differences in the amount of surface area buried when discussing this result. However, if seems a key factor in the stability of the linear filaments would be the direction of the force relative to the complex and attached filament(s), which would be very different for a branch versus a linear filament. The authors should consider addressing this in their discussion.

Fig 4, D-F: It is unclear how the authors determined which filaments were spontaneously nucleated versus those that were nucleated by SPIN90-Arp2/3 complex in these experiments. In reactions containing SPIN90 and Arp2/3 complex what fraction of the filaments will be spontaneously nucleated?

Pg 9 - The observation that VCA negatively influences binding of SPIN90 to the complex is unexpected. What implications does this have for understanding how SPIN90 and VCA synergize to activate the complex?

Fig 4B - Why is there greater nucleation when Arp2/3 complex and GMF are added together compared to renucleation in reactions that don't have any GMF? This is surprising, especially considering that GMF decreases binding of Arp2/3 complex to SPIN90.

Minor Corrections/Comments

Pg 3 "We show that Arp2/3 nucleation is similarly stabilized by cortactin and destabilized by GMF"
Do the authors mean branches and linear filaments nucleated by Arp2/3 complex?

Pg 6- The cyan 3uM data and legend in figure 2B and E is probably too dim to see clearly.

Fig 4 B,C,E,F: It would be best to show the individual data points here if possible.

Pg 16 Please specify which antibody was used to anchor SPIN90.

Referees cross-commenting

I agree with the points that the other reviewers raised.

2. Significance:

Significance (Required)

Comments on significance are in the above section.

3. How much time do you estimate the authors will need to complete the suggested revisions:

Estimated time to Complete Revisions (Required)

(Decision Recommendation)

Between 1 and 3 months

4. *Review Commons* values the work of reviewers and encourages them to get credit for their work. Select 'Yes' below to register your reviewing activity at Web of Science Reviewer Recognition Service (formerly Publons); note that the content of your review will not be visible on Web of Science.

Web of Science Reviewer Recognition

No

Full Revision

Manuscript number: RC-2022-01502
Corresponding author(s): LuYan CAO, Antoine JEGOU, Guillaume ROMET-LEMONNE

1. General Statements [optional]

This section is optional. Insert here any general statements you wish to make about the goal of the study or about the reviews.

We thank the reviewers for their positive evaluation of our manuscript and for their constructive comments which have helped us improve the manuscript. We have addressed all their concerns, as detailed in our point-by-point answer.

The main changes in our manuscript are the following:
- we have added new data in Fig 1C, Fig 3, Fig 4, Fig 5, and we provide new supplementary figures (S2, S4C and S4D, S9).
- In particular, we have performed new experiments with a CA construct from NWASP (Supp Fig S4C and S4D), which provide additional insights into the mechanisms by which VCA destabilizes Arp2/3-nucleated filaments.
- We took this opportunity to repeat our GMF experiments (Fig 3 and Fig 5), which were previously done at T>25°C (as specified), and not at 25°C like all the others. This improves the consistency of our paper: all experiments are now performed at 25°C.
- We now specify the biological repeats of our experiments in figure legends, and show new repeats in Fig 4 and Supp Fig S2.
- We have rewritten parts of the text, in particular the introduction and discussion, in order to integrate the recent structural data from Ding et al. PNAS 2022 (which was not available at the time of our original submission).
- We have slightly modified the title of our manuscript (following a suggestion from Reviewer 1). It is now “Regulation of branched versus linear Arp2/3-generated actin filaments”

To summarize, our paper’s conclusions are unchanged but have been reinforced by additional data, and parts of the manuscript have been re-written to improve clarity.

All the changes in our manuscript are indicated in red.

2. Point-by-point description of the revisions

This section is mandatory. Please insert a point-by-point reply describing the revisions that were already carried out and included in the transferred manuscript.

For clarity, the reviewers' comments are reproduced below, in blue, each followed by our response.

Reviewer #1 (Evidence, reproducibility and clarity (Required)):

An exciting development in our knowledge about how the Arp2/3 complex controls the assembly of actin networks has come from the discovery that in addition to forming branched networks, Arp2/3 can nucleate linear filaments when it is activated by WISH/DIP/SPIN90. However, despite some excellent work largely done by the Nolen lab in yeast, many questions remain about how Arp2/3-mediated assembly of branched vs. linear actin filament. This is especially true in the complex environment of cells, were synergy and competition of different actin networks is used to control biological processes. Knowing the biochemical and physical properties of these different Arp2/3 assemblies will be key to figuring out how they work in cells. Here Cao et al. use an elegant microfluidics based single filament assay system to perform a comparative analysis of the stability of linear and branched Arp2/3 networks. They find interesting differences in how they respond to stabilizing and destabilizing factors. The most striking differences happens when force or aging is applied- both cause debranching of branched networks but have little effect on Spin90-Arp2/3 nucleated filaments.

We thank the reviewer for their positive comments.

Major comments:
As a comparative study on the stability of branched vs. linear Arp2/3 nucleated filaments, this manuscript is fairly complete. The key conclusions are well supported by rigorous experiments which can be reproduced by others based on the information provided. However, I am not seeing explicit information on performing biological replicates. This should be included in the manuscript. The use of statistics is largely fine; however I question the use of one statistical test on one figure (see minor comments below).

The revised manuscript is now explicit about biological replicates. We now specify the biological repeats of all our experiments in the figure legends, and we now show the results from new repeats in Fig 4 and Supp Fig S2 (please see also our response to the minor comments below, for more details).

I would not ask for additional experiments at this time. However, there is an analysis that would be important for interpreting the authors' claims- branch/filament length at the time of dissociation or destabilization of Arp2/3. This would help address if there was a physical tipping

point for each type of structure that could explain potential differences they see. The authors should already have this data and the time to complete it would be negligible in delaying publication.

If we understand correctly, the “physical tipping point” mentioned by the reviewer would be a threshold force, where the Arp2/3-filament interface would become unstable. This is an interesting idea. Indeed, the applied force scales with the length of the filament (or branch), as well as with the flow velocity. In most of our experiments, however, the force applied to SPIN90-Arp2/3 and to branch junctions was kept constant and below 0.2 pN. This was done by exposing the filaments (or branches) to G-actin at the critical concentration, in order to minimize variations of their lengths. Therefore, by design, dissociation events in these experiments take place at the same length, ruling out the existence of a “tipping point”.

Our data provide another test of the reviewer’s hypothesis, thanks to the experiments where we specifically address the question of the impact of force (Fig 5 and Supp Fig S6), by varying length and flow rate. We found that the stability of SPIN90-Arp2/3 linear filaments was unaffected by force, and that debranching was steadily accelerated by force. In both cases, it thus appears that there is no detectable threshold.

One additional major comment is that the manuscript's title and abstract hint that this paper explores the differences in nucleation of branched vs. linear filaments by Arp2/3. However, the only figure that deals explicitly with nucleation in the paper is Figure 1, which is really just a confirmation that the mammalian proteins used in this study perform similarly to their yeast homologues (Balzer et al, Current Biology 2019). The authors might think about rewording the title/abstract to better reflect that paper really explores the differences in the stability of the two networks

This is a fair point. We have now modified the title into “Regulation of branched versus linear Arp2/3-generated actin filaments”.

Minor comments:
1 in 12 men and 1 in 200 women are red/green colorblind. Please change the coloring of the schematics and images so that they can be easily seen by all people. This is especially true of the schematics, which are important for understanding exactly what each assay is measuring.

We thank the reviewer for pointing this out. We have now made the schematics and images in Figs 1A, 2A, 2D and 4D colorblind-friendly.

The Introduction is a bit choppy and unfocused. It was difficult to deduce exactly where the paper was going from it. Please consider re-writing it for better clarity. The Discussion on the other hand was fantastic. Great job on interpreting your results in a larger context.

We have re-written large parts of the Introduction to make it clearer.
We are glad the reviewer liked the Discussion, where we have nonetheless made some small changes in response to comments from the other reviewers.

Many figures- while the use of different lightness values of the same color is appreciated in conveying different concentrations of reagents used, there were several instances where it was very hard to read the one on the very bottom (ex. 2B, E; 3A; 5C, G).

We have now changed the colors in these figures, to make them clearer.

Figure 1- since this is a confirmation of previous results performed using the same proteins from other species, the title should reflect that (ex. VCA domains accelerate the nucleation of filaments by mammalian SPIN90-Arp2/3). Also, to me this figure is supplementary to the main message of the paper. The authors might think of moving it to Supplementary Information.

We have modified the title of Figure 1, now specifying "mammalian", following the reviewer's suggestion. However, we prefer to keep this figure as a main figure, rather than move it to Supplementary as proposed. Indeed, this figure does more than simply confirm previous results with mammalian proteins, since it compares different VCAs, which is new. These results are important because they are put in perspective with our results on the acceleration of linear filament detachment by different VCAs, later in the manuscript.

Figure 1- If the goal was to verify that G-actin recruitment by VCA was important for Spin90-Arp 2/3 nucleation by performing a competition experiment with profilin, why was the concentration of G-actin AND profilin increased between the experiments in 1B vs. 1C. It makes it hard to directly compare the results.

We now provide new data in Fig 1C, which can be directly compared to Fig 1B (only the profilin concentration was increased). It clearly shows that the effect of VCA disappears when the profilin concentration is increased.

Figure 4B-F- Here, it would be nice to see the distribution of all the individual results, which are hidden by the bar graph. Additionally, the Chi-square test is not the appropriate test for evaluating statistical significance between multiple groups. ANOVA followed by an appropriate post hoc test should be used here.

We now show the individual results in the bar graphs of figure 4. In this situation, we agree that the statistical significance should not be evaluated by a Chi-square test. We now indicate the p-values obtained from a paired t-test, which seems appropriate since we are comparing averages in pairs.

Figure 4G- Please quantify and show reproducibility.

We now show quantified repeats (shown in Fig 4, new panels H and I).

Figure 5- the piconewton forces used for these experiments is in line with measured forces that are applied to actin in cells (ex. Mehida et al, Nature Cell Biology 2021; Jiang et al, Nature 2003). The text would benefit if this was explicitly stated.

We now state this explicitly, when presenting these results.

Reviewer #1 (Significance (Required)):

The real significance of this work is in characterizing the differential stabilities of linear vs. branched Arp2/3 filaments in response to actin-binding proteins, mechanical stress, and aging. While both types of filaments respond similarly to actin-binding proteins, with nuanced differences, the most striking results came from applied force and aging experiments, with Spin90-Arp2/3 filaments being much more resistant to both. This has some very interesting implications for how these two types of assemblies might synergize in cells. Additionally, the results also have some exciting implications for the pointed-end regulation of actin filaments, which is still poorly understood in complex systems. Since the manuscript is A) more of a survey study on the factors that influence filament stability that does not go particularly deep into any particular mechanism of regulation and B) has no direct applicability to how the physical properties of branched and linear Arp2/3 nucleated actin filaments influence actin network activity in cells, the audience will likely by limited to actin enthusiasts. However, the work is still important in both what it reveals and implies.

We thank the reviewer for pointing out the novelty and the importance of our work. We agree that the significance of our paper lies in the characterization of the differential stabilities of linear vs. branched Arp2/3 filaments, in response to different physiological factors. One of the strengths of our approach is that we do not focus on one regulatory mechanism in particular. Rather, we reveal fundamental differences between the Arp2/3-generated filaments and how they can be regulated. Understanding these basic mechanisms is a prerequisite to understand the regulation of entire cytoskeletal networks.

Reviewer #2 (Evidence, reproducibility and clarity (Required)):

The quantitative analysis can be improved. It appears that most of the data results from single experiments, with rate values and errors resulting from fitting of single experiments without repetitions. In Fig. 1C legend (p.5) the authors state "These experiments were repeated three times, with similar results", but the data is not used in the analysis and other experiments do not mention this point. This is particularly important for comparisons among different VCAs that are rather similar in nature. In Fig. 1B. N-WASP is more efficient in nucleating SPIN90-Arp2/3 complex-linear filaments followed by WASP and then WASH. In Fig. 2 B,C, N-WASP is the most effective in dissociating SPIN90-Arp2/3 complex linear filaments followed by WASH and then

WASP. But in Fig. 2 E,F, WASH is by far the most effective in dissociating branches followed by N-WASP and then WASP. Therefore, the conclusion in the Discussion (p.12) "While these regulatory proteins similarly affect branched and linear Arp2/3-generated filaments, they do so with clear quantitative differences" is not supported by quantification. To remedy this problem the authors should include at least 3 repeats of each experiment in data analysis. Also, they could include an analysis of sequence differences among VCAs and discuss how these may correlate with the observed differences. For instance, one WH2 in WASP vs. two in N-WASP.

Indeed, we argue that the two forms of activated Arp2/3 differ in their sensitivity to different VCA motifs, based on how these VCA motifs rank in their ability to destabilize branched and linear filaments (the VCA motifs also rank differently in their activation and co-activation of Arp2/3 to nucleate branches and linear filaments, but this result does not contribute to our discussion of how proteins interact with the activated Arp2/3). Following the reviewer's suggestion, we now show repeats of these experiments (new Supp Fig S2), clearly showing that N-WASP is the most effective in dissociating linear filaments while the differences are milder for dissociating branches, with WASH being at least as effective as NWASP. We now also discuss how this observation could relate to differences in sequence between VCAs (Discussion section and new Supp Fig S9).

Also, please note that, following a suggestion from Reviewer 3, we have now performed experiments with the CA-domains of NWASP (new Supp Fig S4C and S4D), which show that the V-domain plays an important role in debranching but plays no role in destabilizing SPIN90-Arp2/3 at filament pointed ends. These new results reinforce our statement that VCA affects branched and linear Arp2/3-generated filaments differently.

Reviewer #2 (Significance (Required)):

Arp2/3 complex is a 7-protein complex implicated in actin filament nucleation and branching. Arp2/3 complex-nucleated branched networks are found at several locations in cells and are responsible for processes such as cell motility.

Cao et al. compare the effect of several proteins on the filament nucleation activity of Arp2/3 complex, and the stabilization or destabilization of actin filament branches as well as linear actin filaments nucleated by SPIN90-Arp2/3 complex. The proteins tested include the VCA regions of three NPFs (N-WASP, WASP, and WASH) that activate Arp2/3 complex, GMF (a debranching protein) and cortactin (a branch stabilizing protein). For the most part, the study uses a single method, microfluidics-TIRF microscopy.

The main findings are:
1. VCA domains enhance nucleation of linear filaments by SPIN90-Arp2/3 complex in the presence of actin monomers.

2. However, VCA domains can also destabilize existing SPIN90-Arp2/3 complex linear filaments and branches, and this effect depends on the presence of of V-domain (WH2 domain that binds actin monomers).
3. The debranching factor GMF also destabilizes SPIN90-Arp2/3 complex linear filaments. Both GMF and VCA generate free pointed ends by dissociating Arp2/3 complex from pointed ends and SPIN90.
4. SPIN90-Arp2/3 complex linear filaments are less susceptible to force and aging than filament branches.
5. Cortactin stabilizes SPIN90-Arp2/3 complex linear filaments to higher degree than it does branches.

These are novel and very interesting new observations of significant interest to the actin cytoskeleton field. Therefore, I recommend publication of this paper in EMBO J.

We thank the reviewer for their positive evaluation of our work.

I have one recommendation and one suggestion for improvement:
Major:
1. The quantitative analysis can be improved. It appears that most of the data results from single experiments, with rate values and errors resulting from fitting of single experiments without repetitions. In Fig. 1C legend (p.5) the authors state "These experiments were repeated three times, with similar results", but the data is not used in the analysis and other experiments do not mention this point. This is particularly important for comparisons among different VCAs that are rather similar in nature. In Fig. 1B. N-WASP is more efficient in nucleating SPIN90-Arp2/3 complex-linear filaments followed by WASP and then WASH. In Fig. 2 B,C, N-WASP is the most effective in dissociating SPIN90-Arp2/3 complex linear filaments followed by WASH and then WASP. But in Fig. 2 E,F, WASH is by far the most effective in dissociating branches followed by N-WASP and then WASP. Therefore, the conclusion in the Discussion (p.12) "While these regulatory proteins similarly affect branched and linear Arp2/3-generated filaments, they do so with clear quantitative differences" is not supported by quantification. To remedy this problem the authors should include at least 3 repeats of each experiment in data analysis. Also, they could include an analysis of sequence differences among VCAs and discuss how these may correlate with the observed differences. For instance, one WH2 in WASP vs. two in N-WASP.

This comment is identical to the reviewer's first paragraph. We copy our answer here again, for convenience:

Indeed, we argue that the two forms of activated Arp2/3 differ in their sensitivity to different VCA motifs, based on how these VCA motifs rank in their ability to destabilize branched and linear filaments (the VCA motifs also rank differently in their activation and co-activation of Arp2/3 to nucleate branches and linear filaments, but this result does not contribute to our discussion of how proteins interact with the activated Arp2/3). Following the reviewer's suggestion, we now

show repeats of these experiments (new Supp Fig S2), clearly showing that N-WASP is the most effective in dissociating linear filaments while the differences are milder for dissociating branches, with WASH being at least as effective as NWASP. We now also discuss how this observation could relate to differences in sequence between VCAs (Discussion section and new Supp Fig S9).

Also, please note that, following a suggestion from Reviewer 3, we have now performed experiments with the CA-domains of NWASP (new Supp Fig S4C and S4D), which show that the V-domain plays an important role in debranching but plays no role in destabilizing SPIN90-Arp2/3 at filament pointed ends. These new results reinforce our statement that VCA affects branched and linear Arp2/3-generated filaments differently.

Minor:
2. In GST-pull-down experiments (Fig. 4G), the amount of Arp2/3 complex bound is analyzed by Western, which is rather unprecise. Is the amount of Arp2/3 complex so little that it cannot be quantified using regular SDS-PAGE? If that is the case, this would suggest rather low affinity of SPIN90 for Arp2/3 complex. How does this affect the proposed mechanism and experiments in the microfluidics chamber?

Indeed, the amount of pulled-down Arp2/3 is low and difficult to quantify by SDS-PAGE. This is consistent with previous reports which indicate a low affinity of SPIN90 for the Arp2/3 complex (Wagner et al. Current Biology 2013, Balzer et al. eLife 2020). This does not affect our conclusions, which we now confirm by showing quantified repeats of our pull-down experiments (new panels H and I, in Figure 4). In spite of this low affinity, which makes it difficult to saturate SPIN90 with Arp2/3, the SPIN90-Arp2/3 interaction is very stable and allows us to carry out our experiments in the microfluidics chamber over several tens of minutes (as was already the case in our previous study, Cao et al. NCB 2020).

Reviewer #3 (Evidence, reproducibility and clarity (Required)):

Summary:

In this study, Cao and collaborators investigate the biochemical and mechanical differences between branched actin filaments nucleated by WASP-activated Arp2/3 complex and linear actin filaments nucleated by SPIN90-activated Arp2/3 complex. They use TIRF microscopy in a microfluidic chamber to show that the mammalian proteins, SPIN90 and WASP (or N-WASP or WAVE), like their yeast homologues, co-activate Arp2/3 complex to nucleate linear actin filaments. Using the same assays, they find the surprising result that the VCA segment of WASP proteins destabilizes the interaction between SPIN90 and Arp2/3 complex in linear actin filaments nucleated by Arp2/3 complex. They then show that VCA also destabilizes actin filament branches. The remainder of the study explores the influence of branch stabilizing/destabilizing proteins or mechanical stress on the stability of the interaction between

SPIN90 and Arp2/3 complex on the pointed end of the actin filament. They find that like branch junctions, SPIN90-bound Arp2/3 is destabilized at the end of linear filaments by GMF and stabilized by cortactin. However, unlike branch junctions, SPIN90-Arp2/3 complex is not destabilized on filament ends by piconewton forces or by aging. They conclude that SPIN90- versus VCA-activated Arp2/3 complex adopt similar but non-identical conformations.

Overall, the paper is well written and the experiments, which are very challenging, are rigorously executed. The biochemical results are convincing, novel and unexpected. However, the work could be strengthened by more strongly connecting the biochemical observations to biological implications. In addition, there are some interpretations/conclusions that seem somewhat weakly supported, and the authors should consider revising. Nonetheless, given the quality of the work and the importance of the system, this manuscript will appeal to a broad audience.

We thank the reviewer for their positive comments. We have rewritten parts of the Discussion in order to better connect our observations to implications in cells. We address the concerns regarding our interpretations in the point-by-point, below.

Comments on evidence, reproducibility, clarity and significance:

The differences in the stability of SPIN90-Arp2/3 on linear filaments verses branch junctions led the authors to conclude that SPIN90- versus VCA-activated complexes adopt similar yet non-identical conformations. There are two problems with this conclusion:
1) This conclusion rests on the idea that the biochemical differences can only be due to differences in the "ground state" active conformations of the complex. Another possible scenario would be that the active conformations are the same, but the transition state or intermediate state structures within the debranching reactions are different, thus changing the kinetics of the debranching reactions.

We thank the reviewer for this remark, and we agree that conformational differences may also arise in the intermediate states, during dissociation (of the branch from the mother, or of the linear filaments from SPIN90). We now mention this possibility in our Discussion.

2.) There are already structural data showing conformational differences between the Dip1-bound Arp2/3 complex on the end of a linear filament and Arp2/3 complex at a branch junction. While there are some caveats to comparisons of the structures (e.g., the Dip1 structure includes the fission yeast SPIN90 protein (Dip1) and the fission yeast Arp2/3 complex while the branch junction contains mammalian proteins), these data offer much stronger evidence that the active states adopt (somewhat) different conformations than the data presented here.

We agree that the available structural data (in particular, Ding et al. PNAS 2022, which was not yet published when we submitted our manuscript, and which we now cite) provide a clear indication that active Arp2/3 adopts different conformations in branches and linear filaments. We have modified our text to make this point clearer.

The authors make comparisons between the Fäβler branch junction structure and the Shaaban Dip1-Arp2/3-filament structure. The Fäβler branch junction structure is a low resolution structure (9 angstroms) and should be interpreted with caution (see below). A much higher resolution of a branch junction structure was recently solved (Ding et al, PNAS 2022) and should be used for comparisons between the structures.

Ding et al. PNAS 2022 was not yet published when we submitted our manuscript. We now use it to compare the structures of active Arp2/3, and we have modified the text accordingly.

Pg 14 - The authors say differences between ARPC3-Arp2 and ARPC5-Arp2 contacts in the two structures are likely to cause the differences in interactions with GMF and VCA. Two concerns with this statement are: 1.) The basis for the conclusion that the ARPC5-Arp2 contacts are different (in Fäβler, et al.) is not solid (see Ding, et al) and 2.) The analysis is vague. To reasonably conclude that differences in the contacts would influence GMF and VCA interactions would require mapping out the structural connection between the ARPC3-Arp2 interaction site and the GMF or VCA binding sites. If there is no obvious connection between these sites, the conclusion that the differences in the ARPC3-Arp2 interface cause differences in VCA and GMF binding should be far more circumspect.

We have re-written this part of the Discussion section. In light of the new data by Ding et al., we agree with the reviewer that the conclusion that the ARPC5-Arp2 contacts are different is not solid. Our revised text makes it clear that we are not making any claims involving interactions within the Arp2/3 complex. Our point is simply that recent cryo-EM reports indicate conformational differences in Arp2 and Arp3 between the two activated forms of the Arp2/3 complex and that, since the CA-domain of NPFs bind to Arp2 and Arp3, it appears reasonable to make a connection with our results.

Pg 6. "These observations suggest that the ability of VCA to destabilize Arp2/3-nucleated filaments relies on the availability of its V-domain." It's possible that G-actin binding to V blocks the CA from accessing the branch junction. Therefore, it seems important to test whether N-WASP-CA can destabilize Arp2/3-nucleated actin filaments.

We thank the reviewer for this suggestion. We now present results from new experiments performed with the CA-domain of NWASP (new Supp Fig S4C,D). We find that the V-domain participates in the enhancement of debranching, but that it appears to play no role in the destabilization of SPIN90-Arp2/3 from the pointed end. It thus seems that the reviewer's proposal is correct, and that G-actin binding to the V-domain blocks the CA-domain from accessing the branch junction. We now propose this interpretation in the text.

Pg 1 - The authors state that "It thus appears that linear and branched Arp2/3-generated filaments respond similarly to regulatory proteins, albeit with quantitative differences". It is worth

considering if one should make a blanket statement that linear and branched filaments respond similarly to regulatory proteins when they have tested 3 in total.

We have rephrased this sentence. It now reads "… respond similarly to the regulatory proteins we have tested…"

Pg 3 - "More generally, the stability of SPIN90-Arp2/3 at the pointed end, which is important to understand the reorganization and disassembly of actin filament networks, remains to be established." In some ways this statement not quite accurate because Balzer et al previously showed that Dip1-Arp2/3 complex is very stable at the pointed end. Is the question here whether that stability is also conserved in mammalian systems? If so, that should be more directly stated.

We meant that, beyond observing that SPIN90 remains visible at the pointed end for some time (as in Balzer et al.), a lot remained unknown: its lifetime had not been quantified, and its sensitivity to the factors that affect branch junctions (proteins, aging, mechanical tension) had not been studied. We have rephrased the sentence in the manuscript to clarify this point.

The observation that VCA accelerates debranching and SPIN90-Arp2/3 dissociation is very interesting. However, it is uncertain if this biochemical activity has biological relevance, given that once nucleation occurs, Arp2/3 complex will move away from the membrane. While the authors mention in the discussion that debranching by VCA could be relevant when the network is compressed near the membrane, this argument is not particularly strong. Are there ways to strengthen this argument, or find another impact this finding might have on our understanding of Arp2/3 complex regulation?

We now mention another situation where branch junctions could encounter membrane-bound VCA domains: on the dorsal and ventral membrane surfaces of lamellipodia. We now cite the recent Kage et al. J Cell Science 2022 and Mehidi et al. NCB 2021, where WAVE has been observed in lamellipodia away from the leading edge.

The observation that SPIN90+Arp2/3-nucleated filaments are not sensitive to piconewton forces is also very interesting. The authors focus on the differences in the amount of surface area buried when discussing this result. However, if seems a key factor in the stability of the linear filaments would be the direction of the force relative to the complex and attached filament(s), which would be very different for a branch versus a linear filament. The authors should consider addressing this in their discussion.

The orientation of the applied force is an interesting point. In their study on debranching, Pandit et al. (PNAS 2020) report that their results are not affected by the angle of the applied force relative to the mother filament (their Fig S1D). We now specify this in our manuscript, when introducing our results on mechanical tension. Similarly, we found that anchoring SPIN90 to the coverslip surface by its N-terminus rather than its C-terminus, which likely affects the orientation

of the applied force, had no impact on our results (Supp Fig S6A). We have now also added a sentence regarding this aspect in our manuscript, after presenting this result.

Fig 4, D-F: It is unclear how the authors determined which filaments were spontaneously nucleated versus those that were nucleated by SPIN90-Arp2/3 complex in these experiments. In reactions containing SPIN90 and Arp2/3 complex what fraction of the filaments will be spontaneously nucleated?

In our conditions, there is no detectable spontaneous nucleation. In control experiments where we flow in the same concentration of G-actin, in the absence of Arp2/3 or in the absence of SPIN90, we observe no filaments at all on the surface, over several fields of view, after 5 minutes. We now specify this in the Methods section.

Pg 9 - The observation that VCA negatively influences binding of SPIN90 to the complex is unexpected. What implications does this have for understanding how SPIN90 and VCA synergize to activate the complex?

It appears that the outcome depends on the context. The main role of VCA during co-activation of the Arp2/3 complex with SPIN90 seems to be to supply G-actin, as already proposed (Balzer, 2020) and confirmed by our results (Fig 1C). In the absence of G-actin, VCA is more likely to remove Arp2/3 from SPIN90 (Fig 4G,I). Similarly, when a filament is already formed, the presence of G-actin mitigates the removal of SPIN90-Arp2/3 from the pointed end by VCA (Supp Fig S4).

Fig 4B - Why is there greater nucleation when Arp2/3 complex and GMF are added together compared to renucleation in reactions that don't have any GMF? This is surprising, especially considering that GMF decreases binding of Arp2/3 complex to SPIN90.

Indeed, there is a small yet statistically significant difference in the re-nucleation fraction we measured in the presence of Arp2/3, with or without GMF (Fig 4B). This may be due to the different timescales of the two situations. In the absence of GMF, the detachment of filaments is slow and new filaments are nucleated from the initial Arp2/3 complexes, which remained bound to SPIN90 upon detachment of the first filaments. In contrast, in the presence of GMF, detachment is faster and accompanied by the departure of the initial Arp2/3, and a fresh Arp2/3 then binds to SPIN90 to nucleate a new filament. It is thus possible that, in the absence of GMF, a small fraction of the SPIN90 and/or their initially bound Arp2/3 complexes would denature over the time they spend at the bottom of the microchamber at 25°C, thereby leading to a slightly smaller re-nucleation fraction. A similar mechanism could be at play in the experiments with or without VCA, in addition to the enhancement of nucleation by VCA (Fig 4C).

Minor Corrections/Comments

Pg 3 "We show that Arp2/3 nucleation is similarly stabilized by cortactin and destabilized by

GMF"
Do the authors mean branches and linear filaments nucleated by Arp2/3 complex?

Yes, that is what we meant. This sentence has now been modified.

Pg 6- The cyan 3uM data and legend in figure 2B and E is probably too dim to see clearly.

The colors have been changed to improve readability.

Fig 4 B,C,E,F: It would be best to show the individual data points here if possible.

We now show individual data points in all these figure panels.

Pg 16 Please specify which antibody was used to anchor SPIN90.

The antibodies are Anti-GST for Nter anchoring of GST-SPIN90, and anti-His for Cter anchoring of SPIN90-His. We now specify this in the Methods section.

CROSS-CONSULTATION COMMENTS
I agree with the points that the other reviewers raised.

Reviewer #3 (Significance (Required)):

Comments on significance are in the above section.

1st Editorial Decision 24th Nov 2022

Thank you for submitting your revised Review Commons manuscript to The EMBO Journal. Based on our editorial assessment of the novelty and interest of the study and the generally positive reviewer comments, I have sent your manuscript back to the original referees.

We have now received a full set of referee comments. I am glad to say that the reviewers find that their previous concerns have been addressed and now recommend publication of the manuscript. I will therefore be happy to accept the manuscript for publication in The EMBO Journal after its reformatting along the guidelines included in the attached document.

Please feel free to contact me if you have any further questions regarding this final editorial revision. You can use the link below to upload the revised files.

Thank you for the opportunity to consider your work for publication, and I look forward to receiving your revised manuscript.

Referee #1:

The authors have sufficiently addressed my concerns and those of the other reviewers from the previous submission. I no longer have any significant concerns and now recommend this article for publication in The EMBO Journal. I very much enjoyed the revised manuscript, which I anticipate will be an important and well received addition to the cytoskeleton field. Excellent job on the resubmission!

Referee #2:

I have now had a chance to evaluate the revised manuscript and data and I am happy to state that this manuscript is now suitable for publication in EMBO J

Referee #3:

The authors have addressed all of my concerns and I support publication.

We thank the reviewers for their positive evaluation of our work.

1st Revision - Editorial Decision 1st Mar 2023

Thank you for addressing the final editorial issues. I am now pleased to inform you that your manuscript has been accepted for publication in the EMBO Journal.

EMBO Press Author Checklist

Corresponding Author Name: Guillaume Romet-Lemonne	
Journal Submitted to: EMBO JOURNAL	
Manuscript Number: EMBOJ-2022-113008	

USEFUL LINKS FOR COMPLETING THIS FORM
The EMBO Journal - Author Guidelines
EMBO Reports - Author Guidelines
Molecular Systems Biology - Author Guidelines
EMBO Molecular Medicine - Author Guidelines

Reporting Checklist for Life Science Articles (updated January
This checklist is adapted from Materials Design Analysis Reporting (MDAR) Checklist for Authors. MDAR establishes a minimum set of requirements in transparent reporting in the life sciences (see Statement of Task: 10.31222/osf.io/9sm4x). Please follow the journal's guidelines in preparing your
Please note that a copy of this checklist will be published alongside your article.

Abridged guidelines for figures
1. Data
The data shown in figures should satisfy the following conditions:
- the data were obtained and processed according to the field's best practice and are presented to reflect the results of the experiments in an accurate and unbiased manner.
- ideally, figure panels should include only measurements that are directly comparable to each other and obtained with the same assay.
- plots include clearly labeled error bars for independent experiments and sample sizes. Unless justified, error bars should not be shown for technical
- if n<5, the individual data points from each experiment should be plotted. Any statistical test employed should be justified.
- Source Data should be included to report the data underlying figures according to the guidelines set out in the authorship guidelines on Data

2. Captions
Each figure caption should contain the following information, for each panel where they are relevant:
- a specification of the experimental system investigated (eg cell line, species name).
- the assay(s) and method(s) used to carry out the reported observations and measurements.
- an explicit mention of the biological and chemical entity(ies) that are being measured.
- an explicit mention of the biological and chemical entity(ies) that are altered/varied/perturbed in a controlled manner.
- the exact sample size (n) for each experimental group/condition, given as a number, not a range;
- a description of the sample collection allowing the reader to understand whether the samples represent technical or biological replicates (including how many animals, litters, cultures, etc.).
- a statement of how many times the experiment shown was independently replicated in the laboratory.
- definitions of statistical methods and measures:
  - common tests, such as t-test (please specify whether paired vs. unpaired), simple χ2 tests, Wilcoxon and Mann-Whitney tests, can be unambiguously identified by name only, but more complex techniques should be described in the methods section;
  - are tests one-sided or two-sided?
  - are there adjustments for multiple comparisons?
  - exact statistical test results, e.g., P values = x but not P values < x;
  - definition of 'center values' as median or average;
  - definition of error bars as s.d. or s.e.m.

Please complete ALL of the questions below.
Select "Not Applicable" only when the requested information is not relevant for your study.

Materials

Newly Created Materials	Information included in the manuscript?	In which section is the information available? (Reagents and Tools Table, Materials and Methods, Figures, Data Availability Section)
New materials and reagents need to be available; do any restrictions apply?	Not Applicable	

Antibodies	Information included in the manuscript?	In which section is the information available? (Reagents and Tools Table, Materials and Methods, Figures, Data Availability Section)
For **antibodies** provide the following information:<br>- Commercial antibodies: RRID (if possible) or supplier name, catalogue number and or/clone number<br>- Non-commercial: RRID or citation	Yes	Materials and Methods

DNA and RNA sequences	Information included in the manuscript?	In which section is the information available? (Reagents and Tools Table, Materials and Methods, Figures, Data Availability Section)
**Short novel DNA or RNA including primers, probes**: provide the sequences.	Not Applicable	

Cell materials	Information included in the manuscript?	In which section is the information available? (Reagents and Tools Table, Materials and Methods, Figures, Data Availability Section)
**Cell lines:** Provide species information, strain. Provide accession number in repository **OR** supplier name, catalog number, clone number, and/**OR** RRID.	Not Applicable	
**Primary cultures:** Provide species, strain, sex of origin, genetic modification status.	Not Applicable	
Report if the cell lines were recently **authenticated** (e.g., by STR profiling) and tested for mycoplasma contamination.	Not Applicable	

Experimental animals	Information included in the manuscript?	In which section is the information available? (Reagents and Tools Table, Materials and Methods, Figures, Data Availability Section)
**Laboratory animals or Model organisms:** Provide species, strain, sex, age, genetic modification status. Provide accession number in repository **OR** supplier name, catalog number, clone number, **OR** RRID.	Not Applicable	
**Animal observed in or captured from the field:** Provide species, sex, and age where possible.	Not Applicable	
Please detail **housing and husbandry conditions**.	Not Applicable	

Plants and microbes	Information included in the manuscript?	In which section is the information available? (Reagents and Tools Table, Materials and Methods, Figures, Data Availability Section)
**Plants:** provide species and strain, ecotype and cultivar where relevant, unique accession number if available, and source (including location for collected wild specimens).	Not Applicable	
**Microbes:** provide species and strain, unique accession number if available, and source.	Not Applicable	

Human research participants	Information included in the manuscript?	In which section is the information available? (Reagents and Tools Table, Materials and Methods, Figures, Data Availability Section)
If collected and within the bounds of privacy constraints report on age, sex and gender or ethnicity for all study participants.	Not Applicable	

Core facilities	Information included in the manuscript?	In which section is the information available? (Reagents and Tools Table, Materials and Methods, Figures, Data Availability Section)
If your work benefited from core facilities, was their service mentioned in the acknowledgments section?	Not Applicable	

Design

Study protocol	Information included in the manuscript?	In which section is the information available? (Reagents and Tools Table, Materials and Methods, Figures, Data Availability Section)
If study protocol has been **pre-registered, provide DOI in the manuscript**. For clinical trials, provide the trial registration number **OR** cite DOI.	Not Applicable	
Report the **clinical trial registration number** (at ClinicalTrials.gov or equivalent), where applicable.	Not Applicable	

Laboratory protocol	Information included in the manuscript?	In which section is the information available? (Reagents and Tools Table, Materials and Methods, Figures, Data Availability Section)
Provide DOI OR other citation details if **external detailed step-by-step protocols** are available.	Yes	Materials and Methods

Experimental study design and statistics	Information included in the manuscript?	In which section is the information available? (Reagents and Tools Table, Materials and Methods, Figures, Data Availability Section)
Include a statement about **sample size** estimate even if no statistical methods were used.	Yes	Figures and figure legends, and source data
Were any steps taken to minimize the effects of subjective bias when allocating animals/samples to treatment (e.g. **randomization procedure**)? If yes, have they been described?	Yes	Materials and Methods
Include a statement about **blinding** even if no blinding was done.	Yes	Materials and Methods
Describe **inclusion/exclusion criteria** if samples or animals were excluded from the analysis. Were the criteria pre-established?<br><br>If sample or data points were omitted from analysis, report if this was due to attrition or intentional exclusion and provide justification.	Yes	Materials and Methods
For every figure, are **statistical tests** justified as appropriate? Do the data meet the assumptions of the tests (e.g., normal distribution)? Describe any methods used to assess it. Is there an estimate of variation within each group of data? Is the variance similar between the groups that are being statistically compared?	Yes	Materials and Methods

Sample definition and in-laboratory replication	Information included in the manuscript?	In which section is the information available? (Reagents and Tools Table, Materials and Methods, Figures, Data Availability Section)
In the figure legends: state number of times the experiment was **replicated** in laboratory.	Yes	Figure legends
In the figure legends: define whether data describe **technical or biological replicates**.	Yes	Figure legends

Ethics

Ethics	Information included in the manuscript?	In which section is the information available? (Reagents and Tools Table, Materials and Methods, Figures, Data Availability Section)
Studies involving **human participants**: State details of **authority granting ethics approval** (IRB or equivalent committee(s), provide reference number for approval.	Not Applicable	
Studies involving **human participants**: Include a statement confirming that **informed consent** was obtained from all subjects and that the experiments conformed to the principles set out in the WMA Declaration of Helsinki and the Department of Health and Human Services Belmont Report.	Not Applicable	
Studies involving **human participants:** For publication of **patient photos**, include a statement confirming that consent to publish was obtained.	Not Applicable	
Studies involving experimental **animals**: State details of **authority granting ethics approval** (IRB or equivalent committee(s), provide reference number for approval. Include a statement of compliance with ethical regulations.	Not Applicable	
Studies involving **specimen and field samples:** State if relevant **permits** obtained, provide details of authority approving study; if none were required, explain why.	Not Applicable	

Dual Use Research of Concern (DURC)	Information included in the manuscript?	In which section is the information available? (Reagents and Tools Table, Materials and Methods, Figures, Data Availability Section)
Could your study fall under dual use research restrictions? Please check biosecurity documents and list of **select agents and toxins** (CDC): https://www.selectagents.gov/sat/list.htm	Not Applicable	
If you used a select agent, is the security level of the lab appropriate and reported in the manuscript?	Not Applicable	
If a study is subject to dual use research of concern regulations, is the name of the **authority granting approval and reference number** for the regulatory approval provided in the manuscript?	Not Applicable	

Reporting

The MDAR framework recommends adoption of discipline-specific guidelines, established and endorsed through community initiatives. Journals have their own policy about requiring specific guidelines and recommendations to complement MDAR.

Adherence to community standards	Information included in the manuscript?	In which section is the information available? (Reagents and Tools Table, Materials and Methods, Figures, Data Availability Section)
State if relevant guidelines or checklists (e.g., **ICMJE, MIBBI, ARRIVE, PRISMA**) have been followed or provided.	Not Applicable	
For **tumor marker prognostic studies,** we recommend that you follow the **REMARK** reporting guidelines (see link list at top right). See author guidelines, under 'Reporting Guidelines'. Please confirm you have followed these guidelines.	Not Applicable	
For **phase II and III randomized controlled trials**, please refer to the **CONSORT** flow diagram (see link list at top right) and submit the CONSORT checklist (see link list at top right) with your submission. See author guidelines, under 'Reporting Guidelines'. Please confirm you have submitted this list.	Not Applicable	

Data Availability

Data availability	Information included in the manuscript?	In which section is the information available? (Reagents and Tools Table, Materials and Methods, Figures, Data Availability Section)
Have **primary datasets** been deposited according to the journal's guidelines (see 'Data Deposition' section) and the respective accession numbers provided in the Data Availability Section?	Not Applicable	
Were **human clinical and genomic datasets** deposited in a public access-controlled repository in accordance to ethical obligations to the patients and to the applicable consent agreement?	Not Applicable	
Are **computational models** that are central and integral to a study available without restrictions in a machine-readable form? Were the relevant accession numbers or links provided?	Not Applicable	
If publicly available data were reused, provide the respective **data citations in the reference list**.	Not Applicable	
